# KMT2C and KMT2D regulate skeletal development through stage-specific epigenetic control of chondrogenesis

**DOI:** 10.1038/s41413-026-00584-8

**Published:** 2026-10-27

**Authors:** Gabrielle Quickstad, Dimitrios V. Bikas, Marielle L. Bond, Sara Vardabasso, Andrew N. Wyper, Young-Kwon Park, Kai Ge, Richard F. Loeser, Terry Magnuson, Karl B. Shpargel

**Affiliations:** 1 Department of Genetics, University of North Carolina, Chapel Hill, NC USA; 2 Department of Cell Biology and Physiology, University of North Carolina, Chapel Hill, NC USA; 3 Laboratory of Endocrinology and Receptor Biology, National Institute of Diabetes and Digestive and Kidney Diseases, National Institutes of Health, Bethesda, MD USA; 4 Thurston Arthritis Research Center, University of North Carolina, Chapel Hill, NC USA; 5 Department of Physiology and Pharmacology, College of Veterinary Medicine, University of Georgia, Athens, GA USA

**Keywords:** Bone, Pathogenesis

## Abstract

Many craniofacial disorders linked to mutations in enhancer-associated chromatin modifiers, including Kabuki syndrome (KS), present with skeletal abnormalities. KS is a craniofacial developmental disorder characterized by mutations in KMT2D, a histone H3 lysine 4 (H3K4) methyltransferase. The cellular origins and molecular pathways leading to KMT2D-dependent skeletal deficits in KS are not well characterized. We previously demonstrated that KMT2D deletion in mouse cranial neural crest cells (cNCCs), multipotent stem cells contributing to craniofacial structures, impairs chondrocyte hypertrophic differentiation. Although we observed defects in cNCC endochondral ossification, KMT2D’s broader skeletal role remains unclear and its functional redundancy in skeletal development with its paralog, KMT2C, is understudied. We now find that KMT2D mutation within chondrocyte lineages results in postnatal growth defects. Chondrocytes deficient in both methyltransferases yield severe deficits in bone formation, revealing redundant roles for KMT2C/D in endochondral ossification. These methyltransferases regulate chondrocyte state transitions as hypertrophic differentiation is impaired with loss of KMT2C/D. When both methyltransferases are deleted in stem cell lineages, cNCCs fail to induce factors downstream of SOX9 activation. At the molecular level, H3K4me1 is lost at de novo chondrocyte enhancers essential for skeletal development, accompanied by reduced H3K27ac deposition, indicating KMT2C/D-dependent enhancer priming. We indicate roles for KMT2C/D in facilitating activation of genes downstream of SOX9 and RUNX2 transcription factors. Our findings define a regulatory cascade mediated by chromatin-level modifications to ensure commitment of chondrocyte lineages, expanding the known roles of histone modifiers and offering insights into the epigenetic underpinnings of skeletal dysplasia and related congenital disorders.

## Introduction

Kabuki syndrome (KS) is a craniofacial developmental disorder whereby patients display facial gestalt including a broad depressed nasal tip, high arched eyebrows, and a high-arched or cleft palate.^1^ The disorder can also include the development of multi-organ deficiencies and skeletal abnormalities. A summary of 13 different clinical reports indicated that 55% of patients present with short stature and 88% present with skeletal abnormalities.^2^ Rib and vertebral anomalies have been observed, including sagittal cleft, butterfly vertebrae, and hemivertebrae.^2^ Patients can also present with cleft hand, brachydactyly, and scoliosis, as well as joint hypermobility and dislocations.^1,3–9^ Together, these patient reports confirm that skeletal abnormalities are a defining feature of KS.

KS is associated with mutations in two distinct histone-modifying proteins, KMT2D (MLL4) and UTX (KDM6A), with poorly characterized function.^10^ Causative heterozygous mutations in KMT2D account for 39%-74% of patient cases (KS1) while mutations in UTX (KS2) are less common (3%-6%).^8,9,11–17^ KMT2D is a histone H3 lysine 4 methyltransferase which, along with its paralog KMT2C (MLL3), is responsible for depositing H3K4me1, a chromatin feature enriched at primed enhancers.^18–23^ Although KMT2C mutations have not been described in KS, they are implicated in the intellectual disorder Kleefstra syndrome 2 (KLEFS2), which also presents with craniofacial components and short stature.^24–26^ UTX, a histone H3 lysine 27 demethylase, functions in a complex with KMT2C and KMT2D and is important for the removal of H3K27me3 from poised enhancer regions so the CREBBP/EP300 complex can acetylate this residue.^27^ Although other H3K4 methyltransferases comprise the catalytic subunit of COMPASS (complex of proteins associated with Set1) complexes, UTX only associates with those containing KMT2C or KMT2D.^28^ KMT2D mutations have been implicated in other craniofacial disorders. Some individuals with Cornelia de Lange syndrome (CdLS)-like or CHARGE syndrome-like features carry KMT2D mutations.^29–32^ These shared phenotypic qualities emphasize the importance of KMT2D in craniofacial and skeletal development.

Notably, short stature was observed in 70% of KS patients carrying *KMT2D* mutations compared to only 25% with other mutations,^33^ implicating KMT2D in chondrocyte-driven bone growth.^34,35^ Most bones develop through endochondral ossification, a process that begins with the condensation of mesodermal mesenchyme to specify chondrocytes, the cell type found in cartilage.^36^ These cells enter a terminal differentiation stage called hypertrophy, forming a scaffold for the primary and secondary centers of ossification to form.^37,38^ Zebrafish models of KS have shown that deletion of *kmt2d* is associated with abnormal chondrocyte differentiation^39^; however, mammalian chondrocyte-specific function requires further exploration. Bone biology has been explored in heterozygous *Kmt2d* gene trap mice as loss of KMT2D resulted in expanded growth plates resulting from excessive differentiation and increased zones of proliferative and hypertrophic chondrocytes.^40^ Additional in vitro data indicated excessive chondrocyte differentiation in the absence of KMT2D.

Previously, we used neural crest cell-specific deletion of *Kmt2d* to model human KS facial pathology.^41^ Cranial neural crest cells (cNCCs) are the multipotent stem cells responsible for the development of all anterior facial bone and cartilage. cNCC-derived chondrocytes in the cranial base undergo endochondral ossification, with bidirectional growth from synchondroses as opposed to unidirectional growth plates.^42,43^ In contrast to other studies, we found that cNCC-specific deletion of *Kmt2d* did not alter chondrocyte distributions in resting and proliferative zones within cranial base synchondroses.^41^ Rather, KMT2D mutant chondrocytes failed to reach the terminal hypertrophic stage necessary for bone formation. Chondrocyte terminal differentiation is necessary for the proper development of bone by promoting vascularization, initiating extracellular matrix mineralization, and indirectly and directly allowing for the replacement of cartilage with bone.^44–46^ Thus, we hypothesize that KMT2D and KMT2C play a broader role in skeletal development during cartilage differentiation, explaining the skeletal pathologies seen in KS and KLEFS2 patients.

In the present study, we utilized an in vitro chondrogenic differentiation model as well as chondrocyte- or cNCC-specific mouse deletion of *Kmt2c* and/or *Kmt2d*. Genetic ablation of *Kmt2c/d* disrupts the enhancer landscape and prevents the deposition of H3K4me1 and activation of lineage-specific enhancers during chondrocyte differentiation. These changes correspond with profound defects in chondrocyte specification, differentiation, and skeletal patterning. Transcriptional and genomic data indicate enhancer co-function with SOX9 and RUNX2 transcription factors that regulate chondrocyte specification and hypertrophic differentiation. Our findings provide evidence that expression of both KMT2C and KMT2D is necessary for chondrocyte establishment and differentiation during bone development.

## Results

### KMT2C and KMT2D have partially redundant function in endochondral ossification

**Fig. 1:**
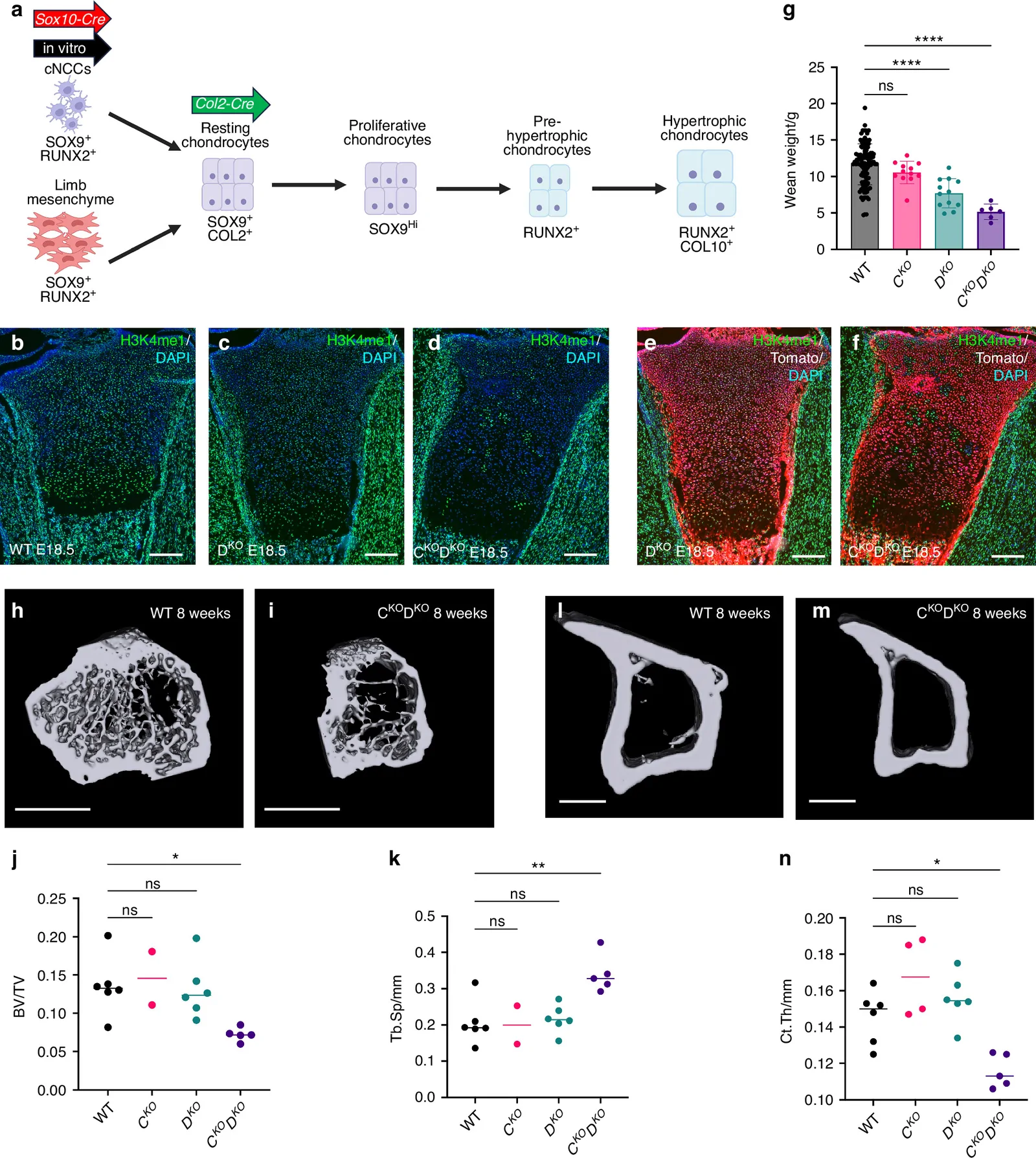
KMT2C and KMT2D play redundant roles in the regulation of bone formation. **a** Model depicting chondrocyte differentiation from both cNCC and limb mesenchyme lineages. *Sox10-Cre* (red arrow), *Col2a1-Cre* (green arrow), and in vitro (black arrow) systems are indicated at the stage of differentiation KMT2C/D loss is occurring in each model. Common markers for each stage of chondrocyte differentiation are labeled. Model created with BioRender.com. Immunofluorescence of coronally sectioned proximal E18.5 tibia in WT, *D*^*KO*^, and *C*^*KO*^*D*^*KO*^ for H3K4me1 and DAPI (**b**–**f**), overlaid with *Rosa*^*Tomato*^ (Tomato) reporter (**e**, **f**). Scale bar is 200 µm. **g** Wean weights (P24) in grams (g) of WT, *C*^*KO*^, *D*^*KO*^, and *C*^*KO*^*D*^*KO*^ (*n* = 112, *n* = 12, *n* = 12, and *n* = 6, respectively). Representative three-dimensional micro-CT reconstructions of 8-week-old WT and *C*^*KO*^*D*^*KO*^ proximal tibial region measured for trabecular (**h**, **i**) and cortical (**l**, **m**) analyses. Transverse view shown in the direction of the distal tibial end. Scale bar is 1 mm (**h**, **i**) or 500 µm (**l**, **m**). **j**, **k** Micro-CT analysis of trabecular bone formation (BV/TV, Tb.Sp) in 8-week-old tibiae of WT, *C*^*KO*^, *D*^*KO*^, and *C*^*KO*^*D*^*KO*^ mice (*n* = 6, *n* = 2, *n* = 6, and *n* = 5, respectively). **n** Cortical thickness in mm shown for WT, *C*^*KO*^, *D*^*KO*^, and *C*^*KO*^*D*^*KO*^ (*n* = 6, *n* = 4, *n* = 6, and *n* = 5, respectively). Data shown are mean ± SD. Statistical analyses were performed with Welch's ANOVA followed by Dunnett’s T3 multiple comparisons test (for wean weight) or ordinary one-way ANOVA followed by Dunnett’s test (for trabecular and cortical analyses). *P < 0.05, **P < 0.01, ***P < 0.001, ****P < 0.000 1, ns = not significant where specified

To dissect the function of KMT2C and KMT2D in endochondral ossification, we generated chondrocyte-specific knockouts by crossing *Kmt2c* and *Kmt2d* conditional alleles with *Col2a1-Cre* transgenic mice (Fig. 1a). *Kmt2c* and *Kmt2d* alleles were engineered with exons floxed upstream or within the catalytic SET domains (Fig. S1a). Prior studies showed that Cre-induced excision of these alleles results in complete loss of KMT2C or KMT2D protein.^41,47,48^ Previous lineage tracing of the *Col2a1-Cre* line demonstrated efficient activity in all cartilage structures by embryonic day 12.5 (E12.5).^49^ This report also described activity within facial mesenchyme and the central nervous system, consistent with *Col2a1* expression in cranial mesenchyme and neuroepithelium. To validate *Col2a1-Cre* specificity, we performed lineage tracing with a *Rosa*^*Tomato*^ reporter allele. Whole-mount imaging of E14.5 embryos showed robust and specific localization of Cre activity to the developing skeleton (Fig. S1b, c). In recombined cells, chondrocyte-specific KMT2D single knockout (*D*^*KO*^; *Kmt2c*^*fl/+*^ *Kmt2d*^*fl/fl*^ *Col2a1-Cre*, see Fig. S1d for genotype abbreviations) produced moderate reduction of H3K4me1 while double knockout of both KMT2C and KMT2D (*C*^*KO*^*D*^*KO*^; *Kmt2c*^*fl/fl*^ *Kmt2d*^*fl/fl*^ *Col2a1-Cre*) was accompanied by marked depletion of H3K4me1 (Fig. 1b–d). Cre efficiency and specificity were confirmed by near uniform activation of the *Rosa*^*Tomato*^ reporter in *D*^*KO*^ and *C*^*KO*^*D*^*KO*^ tibial chondrocytes (Fig. 1e, f).

At birth (postnatal day 0: P0), *C*^*KO*^*D*^*KO*^ mice were significantly underrepresented (8 observed vs. 34 expected), indicative of partial perinatal lethality, whereas genotype frequencies at late embryonic stages (E18.5) remained normal (Fig. S2). Discoloration of *C*^*KO*^*D*^*KO*^ pups at P0 suggested incompletely penetrant lethality due to asphyxia (Fig. S1e). In contrast, *D*^*KO*^ mice displayed milder postnatal growth defects and were born at Mendelian ratios, indicating that a single allele of *Kmt2c* suffices to sustain viability and partially compensate for the loss of KMT2D. Notably, KMT2C single knockouts (*C*^*KO*^; *Kmt2c*^*fl/fl*^ *Kmt2d*^*fl/+*^ *Col2a1-Cre*) was phenotypically comparable to wild-type (WT: Cre-negative littermates) at weaning, supporting the sufficiency of KMT2D in maintaining normal growth in the absence of KMT2C.

We observed pronounced postnatal growth retardation in *C*^*KO*^*D*^*KO*^ mice as well as malocclusion and motor instability at weaning (Figs. 1g and S3a). Importantly, we observed a significant reduction in the overall length of long bones in *D*^*KO*^ and *C*^*KO*^*D*^*KO*^ mice (P0 and 8-week, Fig. S3b, c). *D*^*KO*^ and *C*^*KO*^*D*^*KO*^ mouse weights were normal at birth (Fig. S3d) and displayed recovery by 8 weeks in the instance of tooth overgrowth management (Fig. S3e), suggesting that transient weight loss at weaning (Fig. 1g) is secondary to malocclusion. Therefore, differences in bone size are the primary manifestation of chondrocyte-specific loss of KMT2C/D.

To investigate whether the severe growth phenotypes of *C*^*KO*^*D*^*KO*^ mice were associated with alterations to bone microarchitecture, micro-computed tomography (micro-CT) was performed on skeletally juvenile mice at 8 weeks of age. Analysis of the proximal tibiae revealed that, while WT, *C*^*KO*^, and *D*^*KO*^ exhibited comparable trabecular bone volume fraction (BV/TV), number (Tb.N), thickness (Tb.Th), and separation (Tb.Sp), *C*^*KO*^*D*^*KO*^ mice displayed a marked loss of trabeculation (Fig. 1h, i). Quantitative assessment demonstrated significantly increased trabecular separation and decreased BV/TV, Tb.N, and Tb.Th in *C*^*KO*^*D*^*KO*^ animals relative to controls (Figs. 1j, k and S3f, g). Similar reductions in cortical thickness were found in the tibial midshaft (Fig. 1l–n). These findings implicate combined loss of KMT2C and KMT2D in the pronounced impairment of trabecular and cortical bone formation.

### KMT2C/D mutant growth plates have deficient columnar organization and hypertrophy

**Fig. 2:**
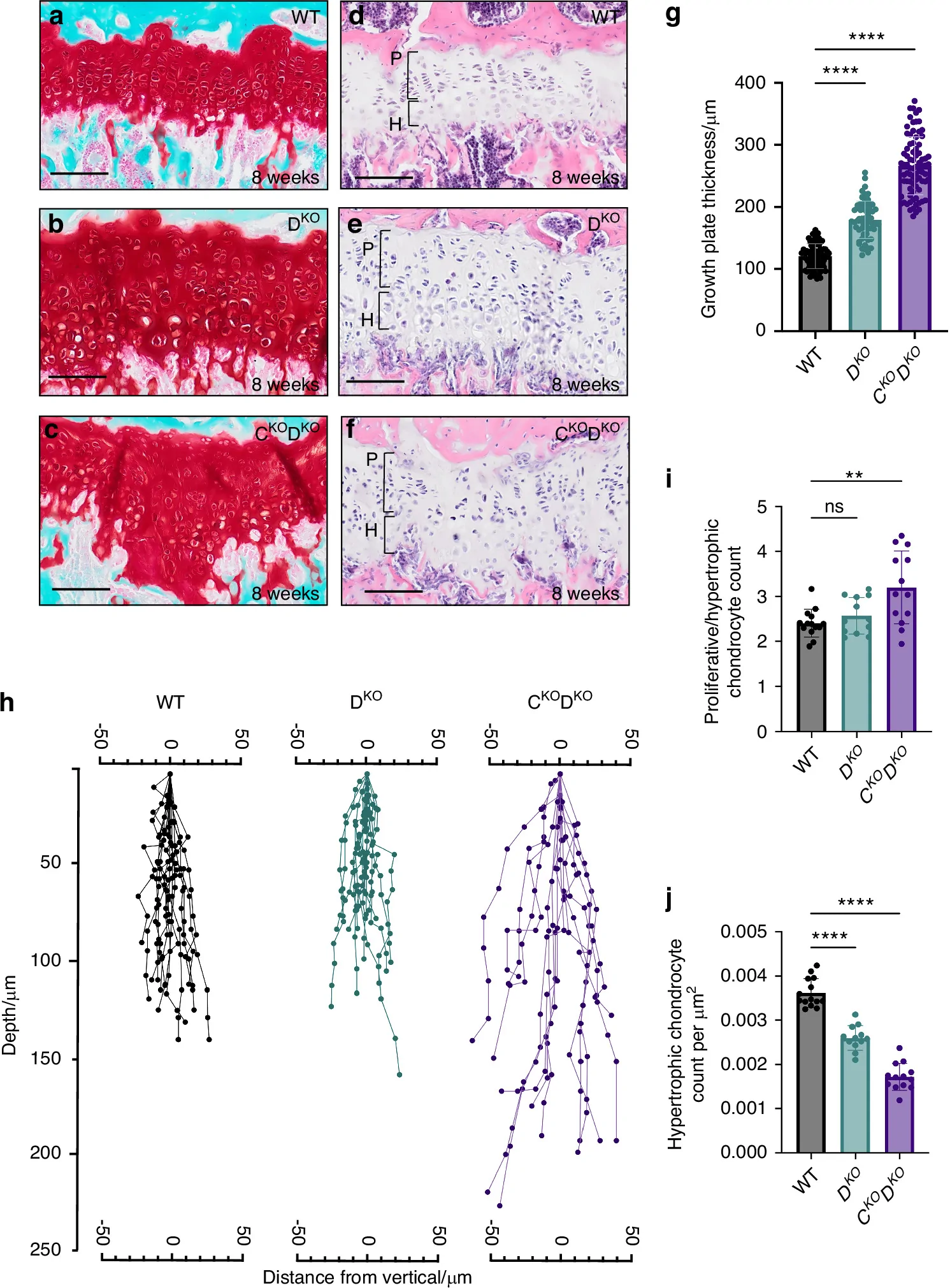
KMT2C/D loss impairs chondrocyte hypertrophy and bone formation. Safranin O/fast green FCF (**a**–**c**) and hematoxylin and eosin (H&E) (**d**–**f**) staining in 8-week-old coronally sectioned proximal tibial growth plates of WT, *D*^*KO*^, and *C*^*KO*^*D*^*KO*^ mice. Proliferative (P) and Hypertrophic (H) regions demarcated by brackets. Scale bar is 100 µm. **g** Eight-week-old proximal tibial growth plate thickness (µm) in WT, *D*^*KO*^, and *C*^*KO*^*D*^*KO*^ (*n* = 4, *n* = 3, and *n* = 4, respectively). **h** Quantitation of longitudinal columns of proximal tibial growth plate chondrocytes measuring ten cells down from the vertical origin point in WT, *D*^*KO*^, and *C*^*KO*^*D*^*KO*^ measured in depth (µm) and distance from the vertical origin point (µm, all *n* = 3). **i**, **j** The ratio of proliferative to hypertrophic chondrocytes and the density of hypertrophic cells per µm^2^ in 8-week-old proximal tibial growth plates of WT, *D*^*KO*^, and *C*^*KO*^*D*^*KO*^ (*n* = 4, *n* = 3, and *n* = 4, respectively). Data shown are mean ± SD. Statistical analyses were performed using ordinary one-way ANOVA followed by Dunnett’s test or unpaired *t*-test with Welch’s correction for *D*^*KO*^ and *C*^*KO*^*D*^*KO*^ comparison. *P < 0.05, **P < 0.01, ***P < 0.001, ****P < 0.000 1, ns = not significant where specified

We performed histological analysis of 8-week coronally sectioned proximal tibial growth plates to determine how loss of KMT2C/D impacted chondrocyte organization and cell morphology. Safranin O/fast green staining revealed a progressively thicker growth plate in *D*^*KO*^ and *C*^*KO*^*D*^*KO*^ tibiae (Fig. 2a–c, g). H&E staining indicated a loss of chondrocyte columnar organization in both proliferative (P) and hypertrophic zones (H) of *C*^*KO*^*D*^*KO*^ (Fig. 2d–f). A spatial representation of the columnar distribution of cells demonstrated a significant loss of linearity in *C*^*KO*^*D*^*KO*^ stacking compared to both WT and *D*^*KO*^ (Fig. 2h). The number of hypertrophic chondrocytes relative to proliferative (Fig. 2i) and the density of hypertrophic cells (Fig. 2j) were reduced in *D*^*KO*^ and more severely impaired in *C*^*KO*^*D*^*KO*^ mice. These altered ratios result from an increase in the total number of proliferative chondrocytes and a decrease in the total number of hypertrophic chondrocytes in *D*^*KO*^ and *C*^*KO*^*D*^*KO*^ mice, as well as an increase in the ratio of proliferative to hypertrophic zone area in *C*^*KO*^*D*^*KO*^ relative to WT (Fig. S4a–c). *D*^*KO*^ and *C*^*KO*^*D*^*KO*^ hypertrophic chondrocyte cell area was increased, suggesting increased vacuolization of late-stage chondrocytes in response to loss of KMT2C/D (Fig. S4d). We also noted zones of safranin O-positive (proteoglycan-expressing) cells expressing chondrocyte markers (RUNX2) embedded within the tibial diaphysis of *C*^*KO*^*D*^*KO*^ mice, indicating that KMT2C/D-deficient chondrocytes may be incapable of effectively transitioning to terminal differentiation and resorption as bone develops (Fig. S4e–g). Altogether, these findings indicate that KMT2C/D-deficient chondrocytes fail to properly organize tibial growth plates and do not undergo normal hypertrophic differentiation.

### KMT2C and KMT2D regulate bone development through chondrocyte hypertrophy

**Fig. 3:**
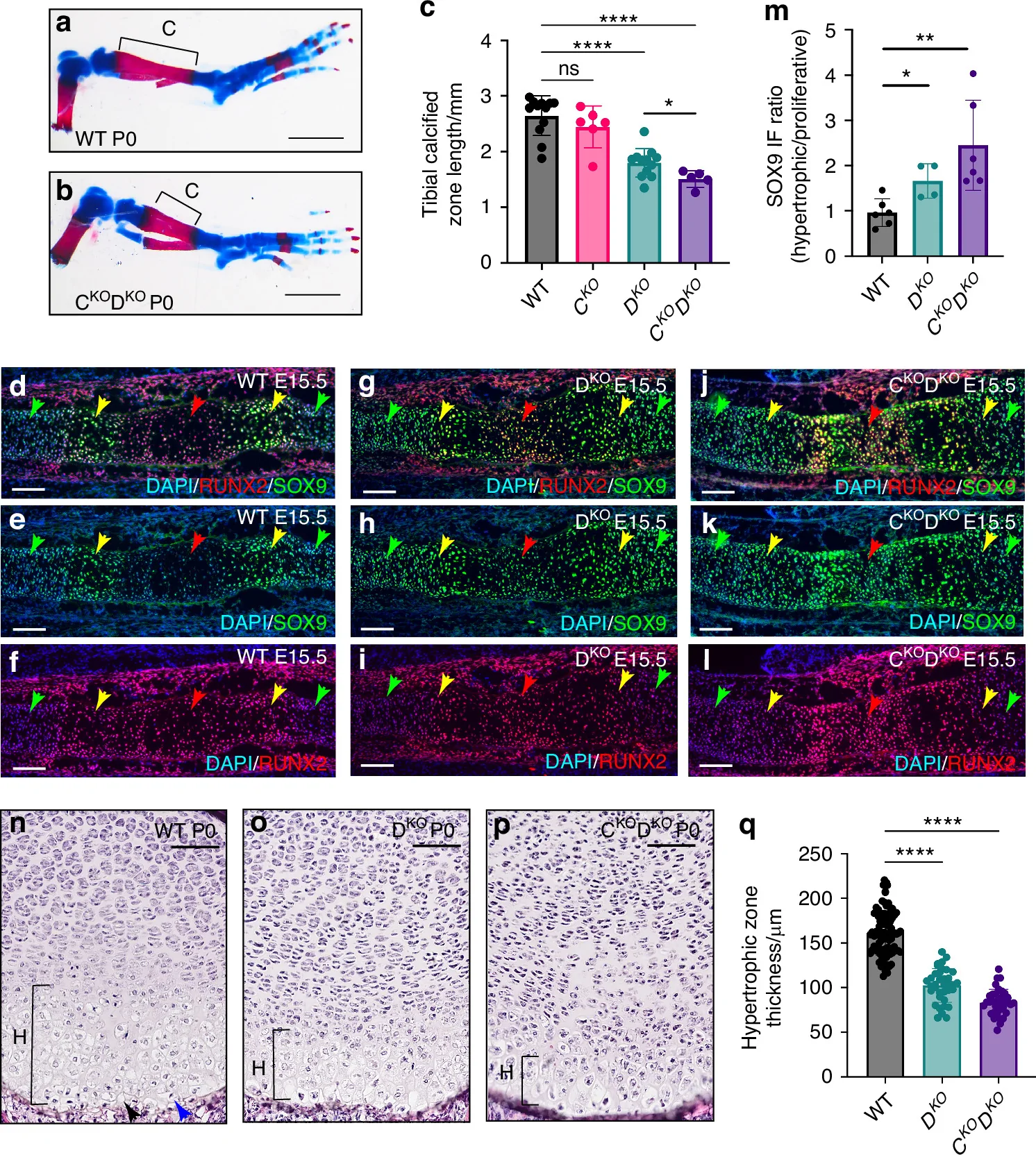
KMT2C/D regulate bone development through chondrocyte hypertrophy. **a**, **b** Alcian blue and alizarin red staining for cartilage and bone of WT and *C*^*KO*^*D*^*KO*^ P0 hindlimbs. Calcified region (C) demarcated with brackets. Scale bar is 1 mm. **c** Length (mm) of calcified region of bone in P0 WT, *C*^*KO*^, *D*^*KO*^, and *C*^*KO*^*D*^*KO*^ (*n* = 12, *n* = 6, *n* = 12, and *n* = 5, respectively). **d–l** Immunofluorescence of SOX9 (green) and RUNX2 (red) in E15.5 tibial primary center of ossification. Arrows demarcate resting (green), proliferative (yellow), and hypertrophic (red) chondrocytes. Scale bar is 100 µm. **m** Quantification of the ratio of SOX9 IF intensity of hypertrophic over proliferative zones in E15.5 WT, *D*^*KO*^, and *C*^*KO*^*D*^*KO*^ (*n* = 6, *n* = 4, and *n* = 6). Data shown are mean ± SD. **n–p** Hematoxylin and eosin (H&E) staining of P0 proximal tibial growth plate in WT, *D*^*KO*^, and *C*^*KO*^*D*^*KO*^. Hypertrophic (H) regions demarcated by brackets. Hypertrophic chondrocytes featuring pyknosis (blue arrowhead) or empty lacunae (black arrowhead) are indicated. Scale bar is 100 µm. **q** Hypertrophic zone thickness (µm) in proximal tibial growth plates of P0 WT, *D*^*KO*^, and *C*^*KO*^*D*^*KO*^ (*n* = 8, *n* = 3, *n* = 3, respectively). Statistical analyses were performed using ordinary one-way ANOVA followed by Dunnett’s test or unpaired *t*-test with Welch’s correction for *D*^*KO*^ and *C*^*KO*^*D*^*KO*^ comparison. *P < 0.05, **P < 0.01, ***P < 0.001, ****P < 0.000 1, ns = not significant where specified

Given the disrupted hypertrophic differentiation in 8-week *D*^*KO*^ and *C*^*KO*^*D*^*KO*^ mice, we hypothesized that an earlier developmental defect might underlie the impaired skeletal growth. Whole-mount skeletal staining of newborn pups (P0) revealed progressive reduction in the length of alizarin red-stained calcified zones within the tibial diaphysis, with the phenotypic severity increasing from *D*^*KO*^ to *C*^*KO*^*D*^*KO*^ relative to WT and *C*^*KO*^ controls (Fig. 3a–c). KMT2C dosage did not affect *D*^*KO*^ phenotypes as bone lengths were indistinguishable on *Kmt2c* wild-type (*C*^*WT*^) or heterozygous (*C*^*HET*^) backgrounds (Fig. S5a–c, g).

In mice, limb bud mesenchymal condensation, chondrocyte specification, and subsequent differentiation occur across E12.5-E15.5, marked by expression of SOX9, a key transcription factor (TF) required for the initiation and maintenance of proliferating chondrocytes.^50^ By E15.5, chondrocytes begin to enter terminal differentiation, characterized by downregulation of SOX9 and upregulation of RUNX2, a TF essential for initiating bone formation.^51,52^ The transition to hypertrophic differentiation is partially regulated by the relative expression and activity of SOX9 and RUNX2.^51,53–63^ E15.5 tibial immunofluorescence (Fig. 3d–l) revealed proper chondrocyte specification in *D*^*KO*^ and *C*^*KO*^*D*^*KO*^, with robust SOX9 expression (Fig. 3h, k). *D*^*KO*^ and *C*^*KO*^*D*^*KO*^ chondrocytes entered proliferation, expressing RUNX2 in pre-hypertrophic chondrocytes (Fig. 3i, l); however, these chondrocytes failed to decrease SOX9 protein levels in primary ossification centers (red arrows in Fig. 3h, k). Quantitative analysis of the ratio of SOX9 protein in hypertrophic relative to proliferative zones indicated a progressive enhancement in *D*^*KO*^ and *C*^*KO*^*D*^*KO*^ embryos (Fig. 3m). E18.5 proximal tibial growth plates revealed sustained *C*^*KO*^*D*^*KO*^ SOX9 expression adjacent to the primary spongiosa (Fig. S6a, b), accompanied by more distal RUNX2 and Osterix (OSX) localization closer to the chondro-osseous junction (Fig. S6c–f). *Col10a1*, a downstream target of RUNX2, displayed consistently reduced expression in *C*^*KO*^*D*^*KO*^ pre-hypertrophic zones (Fig. S6g, h).

To evaluate hypertrophic zone morphology, we performed H&E staining on P0 proximal tibiae (Fig. 3n–p). *D*^*KO*^ and *C*^*KO*^*D*^*KO*^ proximal tibial growth plates exhibited reduced hypertrophic zone thickness as compared to WT (Fig. 3q). Despite the overall decrease in zonal thickness, we noted a significant increase in hypertrophic cell area in both *D*^*KO*^ and *C*^*KO*^*D*^*KO*^ mice at P0 (Fig. S6i). We hypothesized that altered hypertrophy may signify deficient transition to cell death and resorption. Although WT hypertrophic chondrocytes displayed characteristic pyknosis and empty lacunae as these cells transition to terminal differentiation, death, and resorption, *C*^*KO*^*D*^*KO*^ hypertrophic chondrocytes lacked this progression (arrowheads in Fig. 3n and quantification in Fig. S6j). *Kmt2c* and *Kmt2d* were ubiquitously expressed across all chondrocyte cell populations (Fig. S6k–m), suggesting that the observed defects arise from zone-specific regulation of genomic targets rather than differential expression patterns. Overall, these results demonstrate that KMT2C and KMT2D function redundantly to regulate SOX9 downregulation, RUNX2 target activation, and proper transition into chondrocyte hypertrophy.

### KMT2C/D promote hypertrophic differentiation within cNCC and mesodermal chondrocytes

**Fig. 4:**
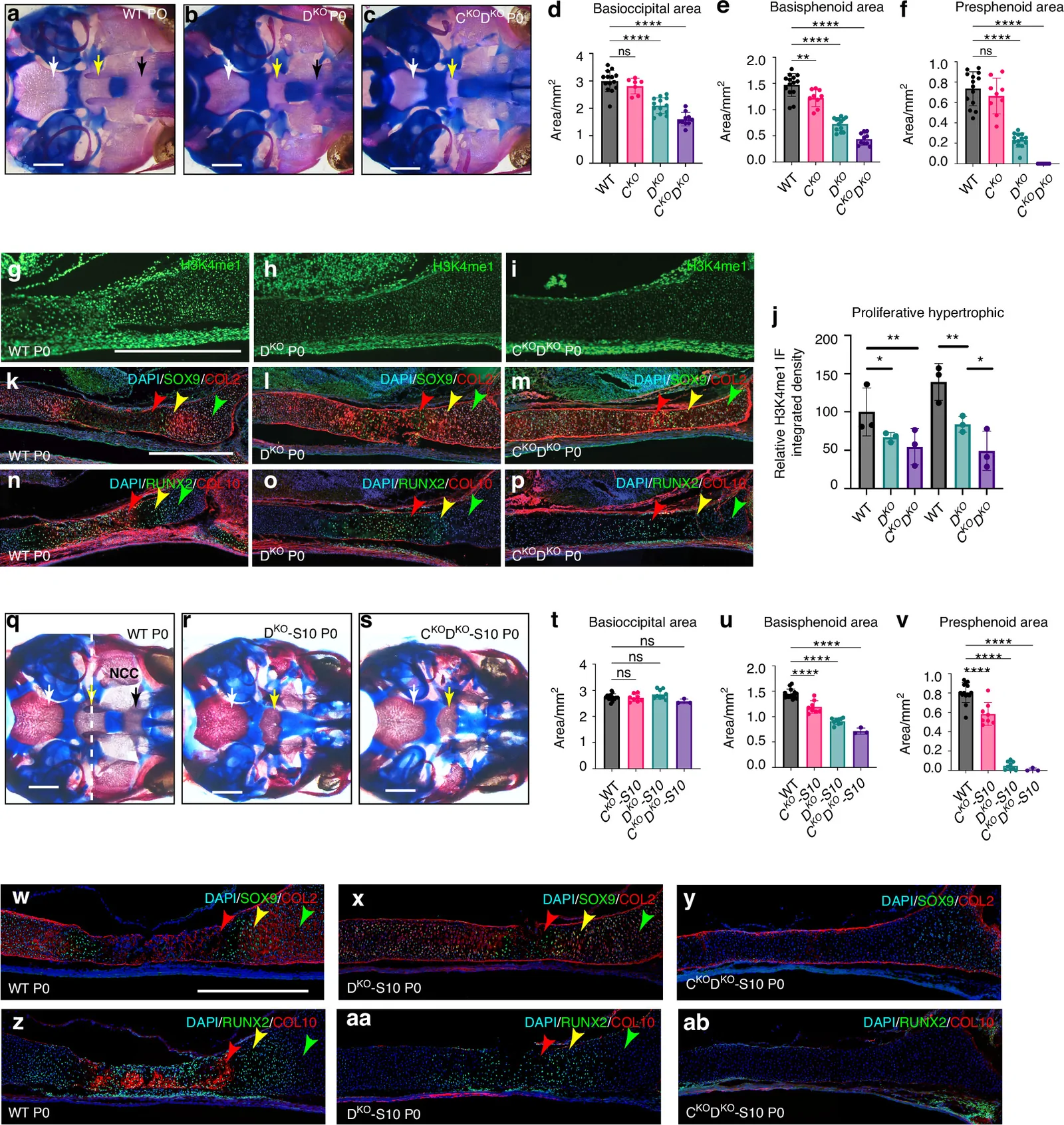
KMT2C/D are required for both chondrocyte specification and maintenance of hypertrophy. **a–c, q–s** Alizarin red and alcian blue staining of P0 ventral whole mount view of the cranial base from *Col2a1-Cre* driven WT, *D*^*KO*^, and *C*^*KO*^*D*^*KO*^ (**a**–**c**) or *Sox10-Cre* driven WT, *D*^*KO*^*-S10*, and *C*^*KO*^*D*^*KO*^*-S10* (**q**–**s**). Basioccipital (white arrow), basisphenoid (yellow arrow), and presphenoid (black arrow) are indicated. Scale bar is 1 mm. In part **q**, cNCC-derived bone formation is specified to the right of the white dashed line (the presphenoid region and anterior basisphenoid region). Scale bar is 1 mm. **d–f, t–v** Area (mm^2^) of basioccipital, basisphenoid, and presphenoid regions of P0 *Col2a1-Cre* (**d–f**: *n* = 14, *n* = 9, *n* = 13, and *n* = 10, respectively) and *Sox10-Cre* (**t–v**: *n* = 14, *n* = 8, *n* = 9, and *n* = 3, respectively) mice. **g–i** H3K4me1 immunofluorescence of sagittal section of the presphenoid region of the cranial base in P0 WT, *D*^*KO*^, and *C*^*KO*^*D*^*KO*^ (Scale bar is 0.5 mm). **j** Relative H3K4me1 integrated density in P0 WT, *D*^*KO*^, and *C*^*KO*^*D*^*KO*^ in both proliferative and hypertrophic zones of the presphenoid region (*n* = 3 for all genotypes). **k–p, w–ab** Immunofluorescence of sagittal section of the presphenoid region in P0 *Col2a1-Cre* driven WT, *D*^*KO*^, and *C*^*KO*^*D*^*KO*^ (**k**–**p**) or *Sox10-Cre* driven WT, *D*^*KO*^*-S10*, and *C*^*KO*^*D*^*KO*^*-S10* (**w**–**ab**) for COL2A1 (red, **k**–**m** and **w**–**y**) and SOX9 (green, **k**–**m** and **w**–**y**) or RUNX2 (green, **n**–**p** and **z**–**ab**) and COL10A1 (red, **n**–**p** and **z**–**ab**) (Scale bar is 0.5 mm). Arrows demarcate resting (green), proliferative (yellow), and hypertrophic (red) chondrocytes. Data shown are mean ± SD. Statistical analyses were performed using ordinary one-way ANOVA followed by Dunnett’s test or unpaired *t*-test with Welch’s correction for *D*^*KO*^ and *C*^*KO*^*D*^*KO*^ comparison. *P < 0.05, **P < 0.01, ***P < 0.001, ****P < 0.000 1, ns = not significant where specified

The cranial base forms through a combination of mesodermal and ectodermal cNCC lineages, with the presphenoid (PS) and anterior half of the basisphenoid (BS) region deriving from cNCCs.^42^ We examined cranial phenotypes in the *Col2a1-Cre* mice to contrast KMT2C and KMT2D function in endochondral ossification within cNCC lineages (hyoid, PS, BS) to those of mesodermal origin (basioccipital = BO, tibia). At birth, *C*^*KO*^*D*^*KO*^ hyoid cartilage showed a total loss of alizarin red staining for calcified zones of bone (Fig. S7a, b). The cranial base in *C*^*KO*^*D*^*KO*^ displayed a dramatic reduction in the areas of the BO, BS, and PS regions of bone with more mild phenotypes in *D*^*KO*^ mice than *C*^*KO*^*D*^*KO*^ (Fig. 4a–f). In *C*^*KO*^*D*^*KO*^, there was a loss of PS bone at birth (Fig. 4c, Fig. S7e, f). Quantification of proliferative and hypertrophic chondrocyte areas in BS and PS regions of *C*^*KO*^*D*^*KO*^ mice indicated a slight reduction in the proliferative region of the PS and a dramatic reduction in the hypertrophic regions of the BS and PS as compared to WT (Fig. S7e–j). KMT2C dosage did not impact *D*^*KO*^ cranial base phenotypes (Fig. S5d–f, h–j) and expression of *Kmt2c* and *Kmt2d* appeared uniform across chondrocyte subpopulations (Fig. S6n–p). Together, these data indicate that both mesodermal and cNCC-derived chondrocytes require KMT2C/D to appropriately stimulate chondrocyte hypertrophy and cranial bone formation.

The failure of chondrocyte differentiation was further exemplified by the persistence of Meckel’s cartilage, a cNCC-derived developmental cartilaginous structure along the mandible, which normally undergoes hypertrophic differentiation late in embryonic development with central regions resorbed by birth.^64^ Meckel’s cartilage persisted in *C*^*KO*^*D*^*KO*^ as compared to WT, indicative of defective terminal chondrocyte differentiation (Fig. S7c, d). This persistence, coupled with the lack of cranial base ossification, suggested a profound systemic failure in the terminal differentiation program. To investigate this at the molecular level, we performed immuofluorescence (IF) on sagittal sections through the cranial base. In the cNCC-derived presphenoid region, both *D*^*KO*^ and *C*^*KO*^*D*^*KO*^ exhibited a progressive loss of H3K4me1 in proliferative and hypertrophic chondrocytes (Fig. 4g–j). Although SOX9 was appropriately activated in chondrocytes within *C*^*KO*^*D*^*KO*^ synchondroses, its downregulation during hypertrophic differentiation was impaired (red arrows in Fig. 4k–m). RUNX2 was activated in pre-hypertrophic chondrocytes; however, downstream targets such as COL10A1 lacked induction (Fig. 4n–p), supporting the tibial observations that loss of KMT2C/D in chondrocytes impedes terminal hypertrophic differentiation.

### KMT2C/D deletion in stem cells restricts initial chondrocyte specification

Since *Col2a1-Cre*-mediated deletion of KMT2C/D occurred after chondrocytes were established from progenitor stem cells, we sought to identify whether these methyltransferases played a role at earlier stages of chondrocyte specification. To address this, we drove *Kmt2c/d* conditional knockout with a *Sox10-Cre* transgene^65^ active within cNCC progenitors of the anterior cranial base (Fig. 1a). Loss of both methyltransferases in *C*^*KO*^*D*^*KO*^*-S10* (*Kmt2c*^*fl/fl*^ *Kmt2d*^*fl/fl*^ *Sox10-Cre*) pups at birth failed to form PS bone and exhibited reduced BS bone dimensions (Fig. 4q–v). Posterior mesoderm lineages such as BO were unaffected by *C*^*KO*^*D*^*KO*^*-S10* mutation (Fig. 4r–t). In a distinct fashion, KMT2D single knockout in cNCCs (*D*^*KO*^*-S10*) induced a greater reduction in PS bone dimensions compared to results utilizing *Col2a1-Cre*, indicating that an earlier loss of KMT2D in the progenitor state may have a greater impact on bone formation than subsequent KMT2D loss in chondrocytes (Fig. 4b, f, r, v).

To examine the chondrocyte cellular state following methyltransferase deletion in cNCCs, we performed IF for markers of resting and differentiated chondrocytes in *Sox10-Cre*-induced mutants (Fig. 4w–ab). In *D*^*KO*^*-S10* newborn pups, SOX9-positive chondrocytes were specified with appropriate expression of type II collagen (COL2 in Fig. 4x). However, loss of COL10A1 indicated a lack of terminal hypertrophic differentiation despite the presence of KMT2C in these chondrocytes (Fig. 4aa). Strikingly, *C*^*KO*^*D*^*KO*^*-S10* mice lacking both methyltransferases expressed minimal levels of SOX9 but did not express COL2 (Fig. 4y), indicating a deficiency in proper chondrocyte specification. Moreover, loss of both methyltransferases abrogated RUNX2 and COL10A1 expression (Fig. 4ab), indicating a failure to initiate hypertrophic differentiation. These findings show that KMT2C/D are critical not only in the transition to hypertrophic differentiation, but also during chondrocyte lineage specification.

### KMT2C/D are required for chondrocyte specification and hypertrophic differentiation in vitro

**Fig. 5:**
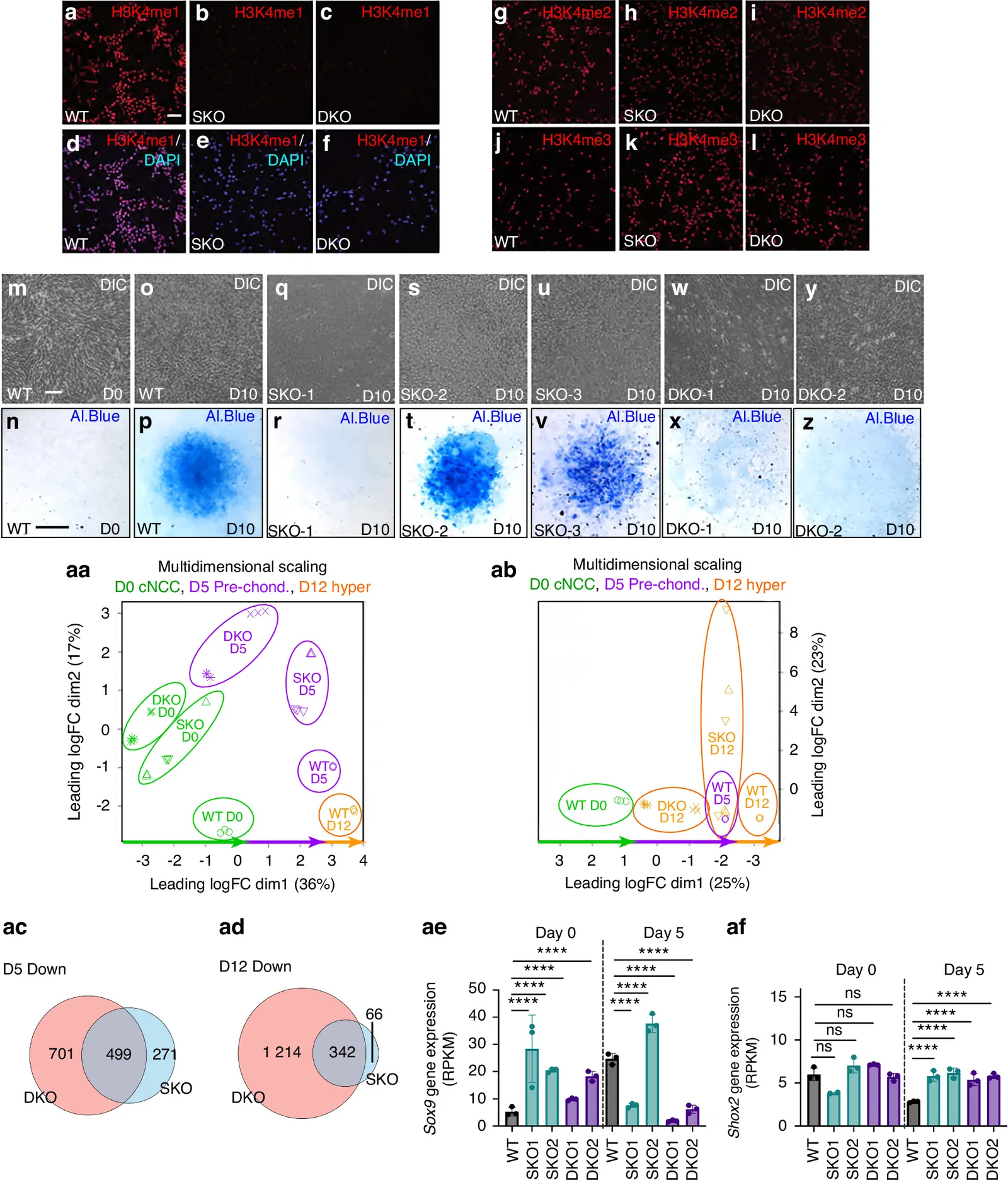
KMT2C/D are required for chondrocyte specification in vitro. **a–l** Immunofluorescence for H3K4me1 (**a**–**f**), H3K4me2 (**g**–**i**), and H3K4me3 (**j**–**l**) in WT, KMT2D SKO, and KMT2C/D DKO lines with or without DAPI (Scale bar is 100 μm). **m–z** DIC imaging and alcian blue staining for proteoglycan production in WT, SKO, and DKO lines. Shown are undifferentiated cNCCs (D0 WT) and day 10 (D10) of chondrogenic differentiation (For **m**, **o**, **q**, **s**, **u**, **w**, **y**, scale bar is 100 um, for **n**, **p**, **r**, **t**, **v**, **x**, **z**, scale bar is 1 mm). **aa, ab** Multidimensional scaling (MDS) plots of WT (circles), SKO line 1 (triangles), SKO line 2 (inverted triangles), DKO line 1 (x), and DKO line 2 (asterisks) in day 0 (D0) cNCCs (green), day 5 (D5) of chondrogenesis (purple), and Day 12 (D12) of hypertrophic differentiation (orange). Corresponding colored arrows display the progression of WT differentiation along dimension 1. **ac, ad** Venn diagrams of downregulated genes in SKO and DKO lines as compared to WT at day 5 (D5) chondrogenic (**ac**) and day 12 (D12) hypertrophic (**ad**) differentiation. **ae, af** RNA-seq gene expression (RPKM) of *Sox9* and *Shox2* at day 0 and day 5 of differentiation in WT, SKO, and DKO lines across all replicates. Data shown are mean ± SD. Statistical significance was determined using false discovery rate (FDR)-adjusted *P*-values derived from differential expression analysis. Asterisks indicate significance based on FDR values: *FDR < 0.05, **FDR < 0.01, ***FDR < 0.001, ****FDR < 0.000 1, ns = not significant

To investigate the temporal dynamics of KMT2C and KMT2D function in gene activation and histone modification, we established an in vitro cellular model of cNCC chondrocyte differentiation (Fig. 1a). We utilized an established primary cNCC stem cell line isolated from E8.5 mouse embryos that retains the potential to differentiate into all cNCC lineages^66^ and features an osteochondral expression profile similar to endogenous cNCCs.^67^ We transfected the cNCCs with a GFP/Cas9/*Kmt2d*-gRNA construct targeting an exon upstream of the mouse floxed allele. Single-cell cloning and sequencing identified two independent lines carrying trans-heterozygous frameshift mutations in exon 49 of *Kmt2d* (single knockout: SKO) (Fig. S8a). To generate double knockout (DKO) lines for KMT2C/D, we transfected SKO lines with *Kmt2c* CRISPR constructs to establish trans-heterozygous mutations in exon 56 of *Kmt2c* (Fig. S8b). Western blot confirmed a loss of KMT2D protein in SKO and DKO lines, and a loss of KMT2C protein in DKO lines (Fig. S9a). Notably, we confirmed that the stability of the H3K27 demethylase UTX is compromised in the absence of KMT2D or the combined loss of KMT2C/D (Fig. S9a) similar to prior reports.^68,69^ *Kmt2c* mRNA was elevated in KMT2D SKO cNCC lines (Fig. S9b, c) but did not impact KMT2C protein levels (Fig. S9a). Both SKO and DKO lines demonstrated marked reductions in H3K4me1 (Fig. 5a–f), a milder decrease in H3K4me2 (Fig. 5g–i), and no significant changes in H3K4me3 (Fig. 5j–l), corroborated by immunoblotting (Fig. S9d). Therefore, KMT2D acts as the predominant H3K4 mono-methyltransferase in these cNCC lines despite similar levels of KMT2C expression (Fig. S9e).

To differentiate cNCCs in vitro, we used an established micromass plating method and cultured cells for 10 days in media supplemented with BMP2 and TGF-β3 (Fig. S10a).^70,71^ At day 10, alcian blue staining of WT micromasses showed robust staining for chondrocyte proteoglycans (Fig. 5m–p), whereas SKO lines had variable staining. The majority of SKO lines (2/3) stained positively for alcian blue (Fig. 5q–v). By contrast, DKO lines uniformly failed to develop alcian blue staining by this time point (2/2 lines) (Fig. 5w–z). Together, these observations demonstrate that loss of KMT2C/D prevents chondrogenic specification in vitro.

To model hypertrophic differentiation in vitro, we maintained cNCC micromasses for 6 days in chondrogenic media, followed by an additional 6 days in hypertrophic media containing T3 (Fig. S10b).^72^ We performed bulk RNA-sequencing and differential expression analyses on WT, SKO, and DKO cNCC lines at undifferentiated (day 0 = D0), chondrogenic (day 5 = D5), and hypertrophic (day 12 = D12) time points (Tables S1–S6). To capture the primary regulatory functions of KMT2C/D, we focused on D5 early chondrogenic stages due to onset of alcian blue staining (Fig. S10c–h). We utilized two independent clones of SKO and DKO lines, including the SKO line that failed to stain for alcian blue during differentiation. Bulk RNA-seq confirmed robust upregulation of canonical chondrogenic (*Sox9* and *Col2a1*) and hypertrophic (Runx2 and *Mmp13*) markers in WT micromasses as previously described (Fig. S10i–l).^73,74^

Multidimensional scaling (MDS) plots of RNA-seq data revealed a progressive increase in transcriptional divergence over the course of WT differentiation (Fig. 5aa, ab). In contrast, D5 SKO micromasses demonstrated an intermediate profile, while D5 DKO closely resembled undifferentiated (D0) WT cultures (Fig. 5aa), indicating that loss of KMT2C/D impedes the initiation of the chondrogenic transcriptional program through redundant mechanisms. At D12 of hypertrophic differentiation, SKO lines did not advance past the WT D5 chondrogenic profile (Fig 5ab), supporting the in vivo data that KMT2D is required for hypertrophic differentiation. Differential gene expression profiles further revealed divergence in downregulated loci between SKO and DKO at D5, while a convergence in SKO and DKO downregulated genes emerged by D12 (Fig. 5ac, ad). These results parallel in vivo observations, where loss of KMT2D alone impaired terminal differentiation and loss of KMT2C/D disrupted both chondrocyte specification and hypertrophy.

Prior literature has shown that promoter-bound KMT2D upregulates *Shox2* within differentiated teratocarcinoma cells, allowing SHOX2 to inhibit *Sox9* to restrict chondrogenesis.^40^ We found that *Sox9* expression was upregulated in SKO and DKO cNCCs at D0 of differentiation and largely became downregulated as compared to WT at D5 (Fig. 5ae). Notably, the variability observed between SKO-1 and SKO-2 D5 *Sox9* expression in our bulk RNA-seq data correlated with micromass proteoglycan expression indicated by presence or absence of alcian blue staining (Fig. 5r, t), suggesting that insufficient *Sox9* may underlie a failure to differentiate in SKO/DKO populations. We did not see significant dysregulation of *Shox2* in SKO or DKO lines prior to differentiation, and in fact saw a mild increase in *Shox2* expression from SKO/DKO lines at D5 (Fig. 5af), which did not appear to correlate with *Sox9* expression patterns or differentiation capacity, suggesting that *Sox9* regulation in cNCC lineages is SHOX2*-*independent.

### Loss of KMT2C/D disrupts RUNX2- and SOX9-driven gene programs governing skeletal system development

To identify molecular pathways underlying impaired bone formation in KMT2C/D mutant mice, we performed Gene Set Enrichment Analysis (GSEA) on a ranked list of all expressed genes from our in vitro RNA-seq. In DKO cells, GSEA revealed significant negative enrichment of the “Skeletal System Development” and “Ossification” gene sets at D5 and D12 of differentiation (NES < -1.6, *q*-value < 0.01) (Fig. S11a–d). The leading-edge genes driving this signal represented key mediators of skeletal processes, including cartilage proliferation, indicating that KMT2C/D loss disrupts essential regulatory networks during skeletal formation.

**Fig. 6:**
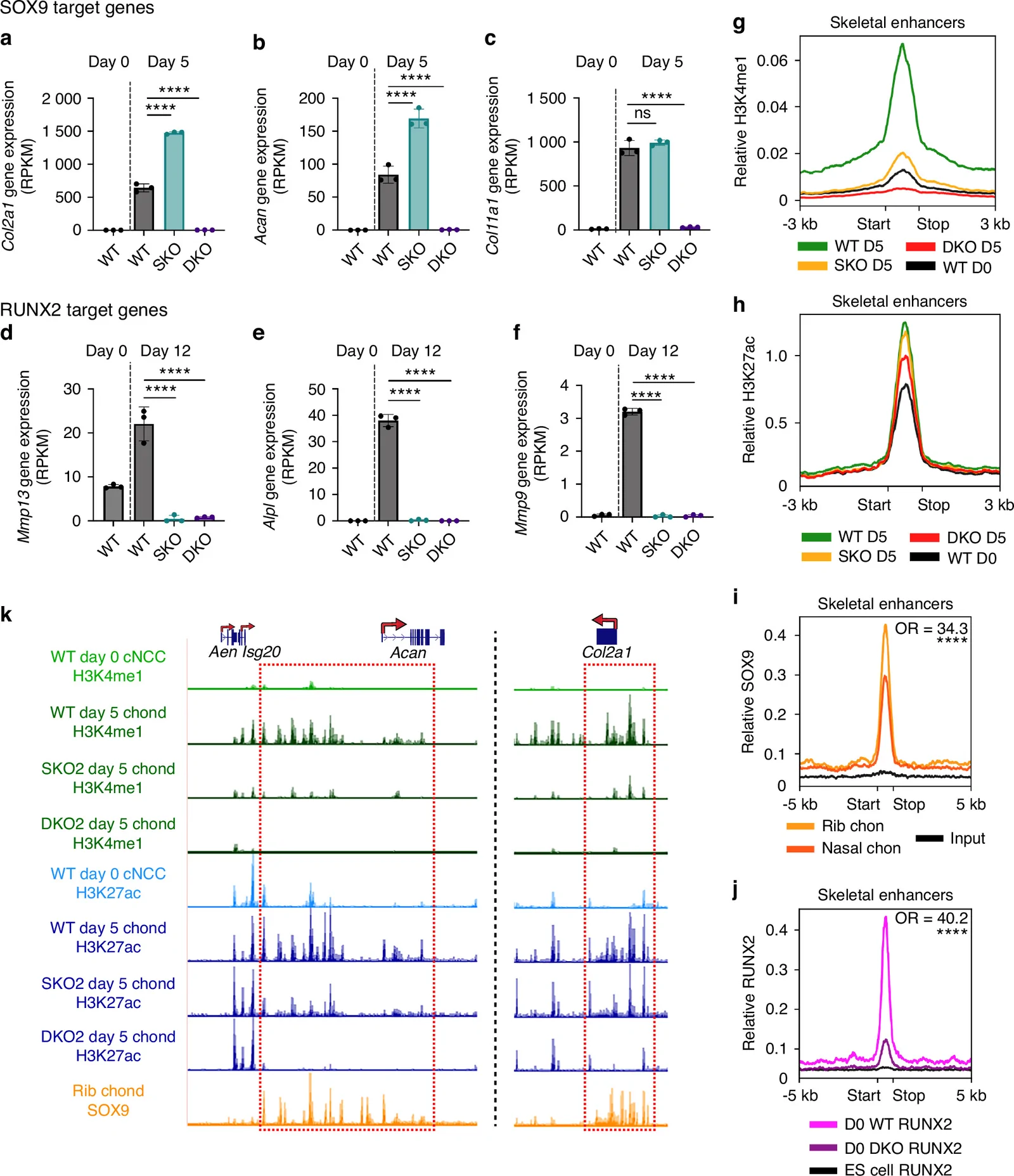
KMT2C/D loss impairs enhancer activity at key regulators of skeletal system development. **a–f** Gene expression (RPKM) of SOX9 (**a**–**c**) and RUNX2 (**d**–**f**) downstream targets at day 5 (**a**–**c**) or day 12 (**d**–**f**) of differentiation in WT, SKO, and DKO lines across all replicates. Plot profiles of H3K4me1 (**g**) or H3K27ac (**h**) levels at WT Day 5 KMT2C/D-regulated skeletogenic enhancers. Illustrated are respective levels at WT day 0 (D0) (black), WT day 5 (D5) (green), SKO day 5 (orange), and DKO day 5 (red). ±3 kb up- and downstream of enhancer peaks. Enrichment of RUNX2 and SOX9 at enhancers associated with skeletal genes downregulated in DKO day 5 cells. **i** SOX9 ChIP-seq from rib chondrocytes (orange), nasal chondrocytes (dark orange), and input controls (black). **j** RUNX2 CUT&RUN in Day 0 WT cNCCs (magenta) compared to DKO cNCCs (purple) and ES cell negative control (black). Odds Ratios (OR) are shown for overlap of SOX9 or RUNX2 with KMT2C/D-regulated skeletal enhancers. **k** UCSC genome browser tracks of enhancer histone modifications H3K27ac (blue) and H3K4me1 (green) in WT day 0 undifferentiated cNCCs (light green or light blue), and WT, SKO, or DKO day 5 differentiated chondrocytes. SOX9 ChIP-seq data from rib chondrocytes (orange) is also shown. Red dashed rectangles demarcate enhancer regions where H3K27ac is lost in knockout lines, correlating with regions of SOX9 occupancy. Gene loci of interest are *Acan* and *Col2a1*. Bar graph data shown are mean ± SD. Statistical significance was determined using false discovery rate (FDR)-adjusted *P*-values derived from differential expression analysis. Asterisks indicate significance based on FDR values: *FDR < 0.05, **FDR < 0.01, ***FDR < 0.001, ****FDR < 0.000 1, ns = not significant (**a**–**f**). Asterisks also denote significance based on two-tailed Fisher’s exact test: *P < 0.05, **P < 0.01, ***P < 0.001, ****P < 0.000 1, ns = not significant where specified (**i**, **j**)

To identify molecular mechanisms whereby KMT2C/D promote chondrocyte specification, we focused on SOX9-regulated pathways and targets given the lack of COL2A1 expression in chondrocytes derived from mutant embryonic cNCCs (Fig. 4y). DKO lines showed significant downregulation of SOX9 targets including *Acan*, *Col2a1*, *Col11a1*, and *Col9a1/2* by D5 of differentiation (Fig. 6a–c, Table S4). In contrast, SKO lines exhibited variable changes as compared to WT. Interestingly, SKO-2, which was capable of proteoglycan expression upon differentiation (Fig. 5t), exhibited elevated levels of SOX9 and downstream targets as compared to WT (Fig. 6a–c). Many KMT2C/D-dependent genes identified in GSEA were characterized as direct RUNX2 targets. At day 12, canonical RUNX2 hypertrophic targets such as *Alpl*, *Mmp9*, and *Mmp13* were significantly downregulated in both SKO and DKO lines (Fig. 6d–f, Tables S5, S6). These transcriptional changes imply that KMT2C/D act on upstream regulatory elements orchestrating both SOX9 and RUNX2 activity. However, given the complexity of the growth plate and the distinct zonal defects observed in histological analysis, it was essential to determine if these molecular disruptions were localized to specific chondrocyte populations in vivo.

To validate our in vitro findings within the context of tissue architecture, we performed spatial transcriptomic analysis on WT and *C*^*KO*^*D*^*KO*^*-S10* cranial bases at birth, with focused analysis of the resting, proliferative, and hypertrophic zones within the PS region (Fig. S12a, b). These zones were delineated by a combination of H&E and nuclear stained cellular morphology as well as RUNX2 and COL10A1 IF. Although resting and proliferative zones showed minimal transcriptomic alteration (Fig. S12c, d), *C*^*KO*^*D*^*KO*^*-S10* hypertrophic chondrocytes displayed dysregulated expression of multiple RUNX2 target genes, including *Col10a1*, *Mmp13*, *Mmp9*, and *Ibsp* (Fig. S12e). GSEA of these spatially defined transcripts also revealed significant negative enrichment of “Skeletal System Development” and “Ossification” gene sets (Fig. S12f, g), consistent with our in vitro findings.

### KMT2C/D loss impairs enhancer activity at key regulators of skeletal development

KMT2C and KMT2D are predominantly responsible for H3K4 mono-methylation in cNCCs and embryonic chondrocytes (Figs. 1d, 4i). To identify direct genomic consequences of KMT2C/D methylation, we performed CUT&Tag profiling of H3K4me1 in D0 cNCCs and D5 chondrocytes in WT, SKO, and DKO lines. H3K4me1 WT peaks of enrichment were dramatically lost at a large set of cNCC targets in both SKO and DKO cells (Fig. S13a). The majority (68%) of WT H3K4me1 peaks localized to intergenic cNCC enhancers rather than transcription start sites, overlapping with previously mapped regions of open chromatin (ATAC-seq) and active histone marks (H3K27ac).^67,75^ We identified KMT2C/D-regulated enhancers as non-TSS (not at transcription start sites) WT peaks that exhibit a significant reduction in H3K4me1 in SKO and DKO lines (Fig. S13b and Table S7). To identify whether KMT2C/D-regulated enhancers were active, we performed CUT&Tag for histone acetylation (H3K27ac). In WT cNCCs, KMT2C/D-regulated enhancers were active with enhanced H3K27ac across chondrocyte specification; however, this chondrogenic enrichment was reduced in SKO and lost in DKO lines (Fig. S13c). Interestingly, a smaller set of novel H3K4me1 enrichment peaks was gained exclusively in DKO cNCCs at genic promoters (61%) within ±1 kb of transcription start sites (Fig. S13a).

To map enhancer-gene regulatory relationships, we applied the Activity-By-Contact (ABC) model,^76^ integrating publicly available Hi-C data^77^ from wild-type mouse cNCCs with our H3K27ac CUT&Tag datasets from D0 and D5 WT and DKO lines. We retained only strong enhancer-promoter pairs (ABC score > 0.02) for downstream analysis. Focusing on genes implicated in “Ossification” and “Skeletal System Development” by GSEA (Fig. S11a, b) that lose expression in DKO at D5, we identified 82 candidate genes linked to 1 030 unique putative enhancers (Table S8). In WT cells, these enhancers exhibited strong activation signatures, with gains in both H3K4me1 and H3K27ac between D0 and D5, consistent with establishing new active enhancer activity during chondrogenic differentiation (Fig. 6g, h). By contrast, DKO D5 samples failed to exhibit any H3K4me1 and showed markedly reduced H3K27ac at the same sites. SKO D5 histone profiles were more intermediate, suggesting partial compensation by KMT2C. We conclude that KMT2C and KMT2D are redundantly priming and activating enhancers to regulate chondrogenic gene expression critical for bone formation.

**Fig. 7:**
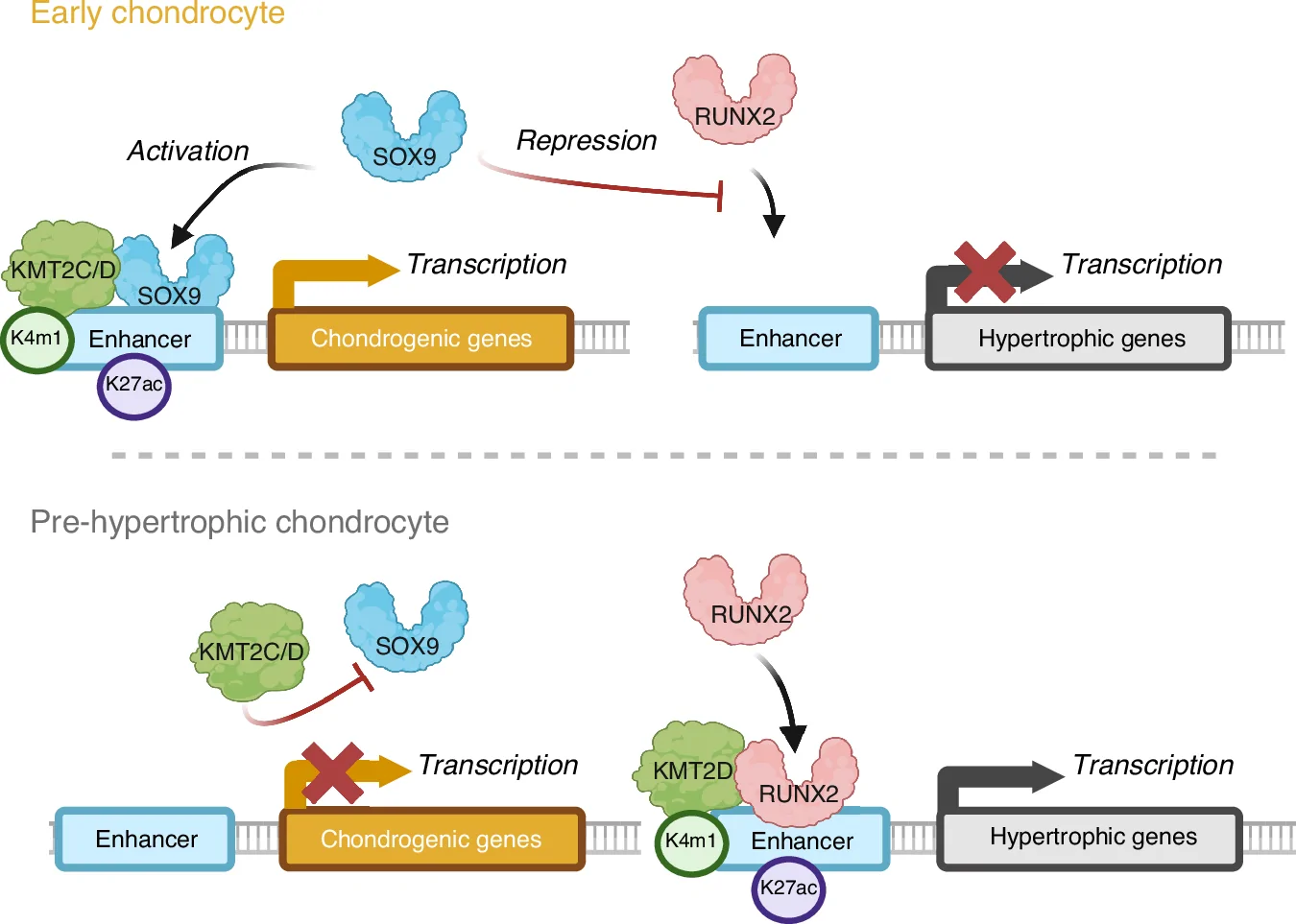
Model illustrating the roles of KMT2C and KMT2D during early chondrocyte specification and the transition to hypertrophic differentiation. (Top) During early chondrogenesis, KMT2C/D facilitate the upregulation of SOX9 protein transcriptional activity. Presence of KMT2C, KMT2D, and SOX9 at enhancers of chondrogenic genes is essential for proper enhancer activation and subsequent transcription of downstream target genes. SOX9 also inhibits RUNX2 by preventing its DNA-binding activity, thereby maintaining the early chondrocyte state. (Bottom) In pre-hypertrophic chondrocytes, KMT2C and KMT2D contribute to SOX9 repression, which permits RUNX2 binding to enhancers of hypertrophic target genes. The presence of KMT2D is required for enhancer activation and RUNX2 recruitment driving hypertrophic differentiation. Model created with BioRender.com

Given the misregulation of RUNX2 and SOX9 downstream targets in SKO and DKO lines, we examined TF occupancy at identified enhancers. We performed RUNX2 CUT&RUN in WT and DKO cNCCs and utilized SOX9 ChIP-seq data from mouse chondrocytes.^78^ Both RUNX2 and SOX9 showed significant enrichment at KMT2C/D-regulated skeletal enhancers (Fig. 6i, j). Fisher’s exact test revealed broader SOX9 occupancy (63.7% of query intervals; 656/1 030; OR = 34.3; *P* < 0.000 1) and higher RUNX2 specificity (OR = 40.2; *P* < 0.000 1) despite smaller overlap (15.8%; 163/1 030). Therefore, KMT2C/D loss may not only impair enhancer activation, but also coordinate SOX9 and RUNX2 occupancy at critical enhancers during endochondral ossification (Fig. 7). UCSC genome browser tracks of SOX9 targets, *Acan* and *Col2a1*, revealed a broad absence of H3K4me1 at enhancers in SKO and DKO lines, but specificity regarding H3K27ac loss (red rectangles in Fig. 6k). Enhancer H3K27ac loss correlated with SOX9 chondrocyte occupancy. CUT&RUN in DKO cNCCs revealed significant (edgeR FDR < 0.05) reduction in RUNX2 occupancy at the majority of D0 peaks (6 884/6 979 peaks, Fig. S13d, Table S9). Similar DKO loss of RUNX2 occurred at KMT2C/D regulated skeletal enhancers (Fig. 6j), correlating with KMT2C/D function (Fig. S13e). These data support a model for cell-type-specific regulation of enhancer activity by KMT2C/D, involving more complex catalytic-independent recruitment of SOX9 or RUNX2 to promote enhancer-regulated transcription.

## Discussion

Prior studies demonstrated that loss of KMT2D and UTX in mesenchymal precursors disrupted differentiation into cartilage- and bone-forming cells in vitro, suggesting a broader role for KMT2D beyond craniofacial development.^79,80^ Expanding on these findings, we now show that KMT2C and KMT2D are critical for endochondral ossification from limb mesenchyme- and cNCC-derived chondrocytes. Our results demonstrate that KMT2C and KMT2D are not functionally equivalent in mammalian development. Chondrocyte-specific deletion of both methyltransferases or *Kmt2d* alone led to marked skeletal defects. In contrast, deletion of *Kmt2c* alone produced no significant abnormalities in skeletal microarchitecture. Loss of both methyltransferases resulted in more severe impairments to cartilage and bone formation than loss of either gene alone. In a similar fashion, prior literature established a partially redundant role for KMT2C and KMT2D in adipose tissue development.^22,47,81^ Collectively, we provide evidence supporting a broader role for KMT2C and KMT2D in skeletal development, indicating that epigenetic regulators may contribute to a wider spectrum of undiagnosed skeletal dysplasia.

We previously demonstrated KMT2D requirement in cNCCs for craniofacial chondrocytes to complete hypertrophic differentiation.^41^ Here, we expand on these findings to contrast how enhancer methyltransferases impact transition of chondrocyte states. Mutation with *Col2a1-Cre* following chondrocyte specification impaired hypertrophic differentiation through dysregulation of RUNX2 target genes. This dysregulation had profound effects on skeletal development, offering a potential mechanism behind pediatric growth disorders. Mutation of the methyltransferases with *Sox10-Cre* in cNCCs prevented chondrocyte lineage specification, evidenced by lack of expression of *Col2a1* and an inability of DKO cNCCs to differentiate in vitro. As cNCC-derived tissues are impacted in craniofacial developmental disorders, these observations link chondrocyte lineage specification to downstream structural anomalies characteristic of KLEFS2 and KS.

*Sox9* expression was reduced during in vitro differentiation in the absence of KMT2C/D. By contrast, in vivo, we observed continued expression of *Sox9* in *D*^*KO*^ and *C*^*KO*^*D*^*KO*^ hypertrophic chondrocytes, implying a failure to transition to RUNX2-dominant hypertrophy, consistent with impaired terminal differentiation. SOX9 directly interacts with RUNX2 to prevent its ability to access target genes.^53^ Accordingly, excess SOX9 may prevent RUNX2-driven gene activation in the absence of KMT2C/D. Extrinsic cues in vivo absent from micromass cultures may account for the discrepancy between *Sox9* expression patterns in vivo and in vitro in the absence of KMT2C/D. Additionally, cNCCs lack KMT2C/D prior to chondrogenic lineage commitment in vitro, whereas the *Col2a1-Cre* gene deletion occurs after specification (Fig. 1a). Despite this discrepancy, we observed consistent downregulation of *Runx2* and downstream target genes by in vitro differential expression analysis, *Sox10-Cre* spatial transcriptomic data, and *Col2a1-Cre/Sox10-Cre* driven *C*^*KO*^*D*^*KO*^ immunofluorescence. Therefore, absence of KMT2C/D disrupts the transition from SOX9- to RUNX2-guided chondrocyte differentiation, though the precise mechanism of KMT2C/D regulation of RUNX2 function warrants further investigation.

Prior studies examined KMT2D function in Kabuki syndrome by assaying promoter H3K4me3.^40,82^ Although we observed global loss of H3K4me1 and a reduction in H3K4me2 upon loss of KMT2D or KMT2C/D, we saw no change in H3K4me3 levels, consistent with the known lack of KMT2D trimethylation activity.^83^ Further investigation of SKO and DKO putative enhancers of downregulated skeletal genes revealed a failure to gain H3K4me1 and H3K27ac upon differentiation, which were normally acquired in WT chondrocytes. SKO lines had milder chromatin and gene expression effects, consistent with partial compensation by KMT2C. SOX9 binding was enriched at KMT2C/D-regulated skeletal enhancers, suggesting KMT2C/D directly facilitate SOX9 occupancy at these sites. KMT2C/D were required for efficient RUNX2 binding to genomic targets. These findings support a model in which KMT2C/D co-function with SOX9/RUNX2 to alter the active enhancer landscape during critical transition points of chondrocyte differentiation to allow for appropriate regulation of downstream skeletal genes (Fig. 7).

In addition to a loss of H3K4me1 at enhancers, we also saw a mild reduction in H3K27ac. Track data of downstream chondrocyte target genes indicated that region-specific acetylation loss corresponded with critical chondrocyte TF binding. This points to a subset of enhancers governing chondrocyte differentiation that are uniquely sensitive to the presence or absence of regulatory factors (i.e., SOX9, RUNX2, and KMT2C/D) (Fig. 7). Alternatively, pronounced disruption of gene expression and a reduction in H3K27ac by loss of KMT2C/D could indicate catalytic-independent function for these methyltransferases. For instance, UTX destabilization resulting from KMT2D deletion could impact H3K27 demethylation at inactive poised enhancers (Fig. S9a).^69^ Furthermore, KMT2C/D catalytic domain mutation had mild effects on gene expression compared to null mutations.^84^ These data suggest that histone modifiers serve additional recruitment and/or regulatory roles at enhancers.

We provide comprehensive in vivo and in vitro models to define the roles of KMT2C and KMT2D at two critical transition points, specification and hypertrophy, in chondrocyte differentiation. Our results point to epigenetic enhancer dysfunction as a mechanism for skeletal malformations and demonstrate redundancy between KMT2C and KMT2D in skeletal development. Differing outcomes from the *Col2a1-Cre* and *Sox10-Cre* deletions emphasize the importance of timing and chondrocyte lineage specificity in KMT2C/D function. Future molecular studies utilizing primary mouse chondrocytes may clarify lineage-specific roles of KMT2C/D and allow direct assessment of SOX9 and RUNX2 chromatin binding dynamics. Together, our data provide evidence for regulation of chondrocyte differentiation by KMT2C/D modulation of enhancer activity, highlighting the need for future research of epigenetic regulators in skeletal development. Such studies will continue to uncover how disruptions in enhancer activity contribute to skeletal dysplasia.

## Materials and methods

### Mice

All mouse experiments were conducted under UNC IACUC-approved protocols. *Col2a1-Cre*,^49,85^ *Sox10-Cre*,^65^ *Kmt2c*^*fl*^ ^48^, *Kmt2d*^*fl*^,^41,81,86^ and *Rosa*^*Tomato*^^87^ reporter lines were used; Cre-negative littermates served as controls throughout.

### Tissue preparation

Embryonic and postnatal limbs were fixed in 4% paraformaldehyde (PFA), processed through sucrose gradients, and cryosectioned (8–10 µm) or paraffin-embedded and sectioned (10 µm) after decalcification. Eight-week hindlimbs were fixed 48 h, decalcified 6 days in Immunocal, and coronally sectioned through the knee joint.

### Histology

Sections were stained with safranin O/fast green,^88^ toluidine blue O,^89^ or H&E^90^ using standard protocols. P0 skeletal preparations were stained with alizarin red S and alcian blue.^91^ Imaging was performed on a Leica SP8X or Evident VS200 slide scanner; quantification used Fiji/ImageJ^92^ and QuPath.^93^ See supplemental methods for details on tissue scoring.

### Immunofluorescence and western blotting

Immunofluorescence was performed as described^94^ with antibodies against SOX9, RUNX2, COL2A1, COL10A1, OSX, UTX, and H3K4me1 (see supplemental methods). Eight-week paraffin sections underwent antigen retrieval and autofluorescence quenching prior to staining. Western blotting,^68,95^ assessed H3K4me1, H3K4me2, KMT2C, KMT2D, and UTX levels from nuclear and histone extractions.^86^

### Micro-computed tomography (micro-CT)

Hindlimbs fixed in 4% PFA were scanned at 12 µm voxel resolution (70 kVp, 114 µA). Trabecular microstructure was evaluated in a 1.2 mm ROI below the proximal growth plate. Cortical measurements (1.2 mm ROI) were assessed 2 mm below the proximal growth plate. Representative three-dimensional micro-CT reconstructions generated using 3D Slicer.^96^

### RNA in situ hybridization

RNA in situ hybridization was performed as previously described.^90^ T7-transcribed digoxigenin-labeled antisense riboprobes were designed from *Kmt2c* and *Kmt2d* regions utilized by the Allen Brain Atlas (https://mouse.brain-map.org) similar to prior experimentation.^41^ *Col10a1* probe corresponded to position chr10:34395357-34395970 within the GRCm38 (mm10) genome.

### Spatial transcriptomics

NanoString GeoMx WTA (19 962 genes) was performed on P0 presphenoid cranial base sections, with ROIs defined by H&E and RUNX2/COL10A1 immunofluorescence to assay gene expression in resting, proliferative, and hypertrophic chondrocyte zones with analyses^97–99^ performed by the UNC Bioinformatics and Analytics Research Collaborative Core.

### Cell culture

O9-1 cranial neural crest cells (cNCCs) were maintained on Matrigel in MEF-conditioned media as described.^66^ KMT2D single (SKO) and KMT2C/D double knockout (DKO) clonal lines were generated by LentiCRISPRv2GFP (see supplemental methods). Chondrogenic differentiation used the micromass method^70,71^ for 10 days as described^66,100^; hypertrophic differentiation extended to day 12 with T3/β-glycerophosphate media.^72^

### RNA-seq

Triplicate RNA libraries from WT, SKO, and DKO lines (undifferentiated and differentiated) were generated with the KAPA mRNA HyperPrep Kit, sequenced (NovaSeq X, 50 bp paired-end), and analyzed as described in the supplemental methods.

### CUT&Tag and CUT&RUN

H3K4me1 and H3K27ac CUT&Tag and RUNX2 CUT&RUN were performed on cNCCs at multiple differentiation stages using Epicypher pAG-Tn5/pAG-Mnase protocols as described.^67^ K-MetStat and *E. coli* spike-in normalization were applied with analyses as described in the supplemental methods.

### Activity-By-Contact (ABC) model

Activity-By-Contact model was applied as described^76^ with adaptations^101^ to identify enhancer-gene pairs using H3K27ac CUT&Tag peaks as candidate enhancers and publicly available cNCC Hi-C data.^77^ Enhancer-gene pairs with ABC score > 0.02 and within a 5 Mb window were retained. Skeletal enhancers were defined by overlap with GSEA ossification/skeletal development gene sets; SOX9/RUNX2 binding enrichment was assessed by Fisher’s Exact Test.

### Statistics

Analyses were performed in GraphPad Prism 10. One-way ANOVA with Dunnett’s test, Welch's ANOVA with Dunnett’s T3, or Welch’s unpaired *t*-test were used as appropriate. Data are mean ± SD; *P*<0.05 was considered significant.

## Supplementary information

Supplementary Methods

Combined Supplementary Figures S1-S13

Supplementary Table S1

Supplementary Table S2

Supplementary Table S3

Supplementary Table S4

Supplementary Table S5

Supplementary Table S6

Supplementary Table S7

Supplementary Table S8

Supplementary Table S9

## Data Availability

RNA-seq and CUT&Tag datasets are in submission to FaceBase (accession FB00001420): https://doi.org/10.25550/8C-WDXG.
