# Supplementary material for: KMT2C and KMT2D regulate skeletal development through stage-specific epigenetic control of chondrogenesis
Source: Bone Res. 2026 Oct 27;14:107. doi: 10.1038/s41413-026-00584-8 (PMC13714318; doi:10.1038/s41413-026-00584-8)
Supplement: Supplementary file 1 [file 41413_2026_584_MOESM2_ESM.pdf]

Figure S1

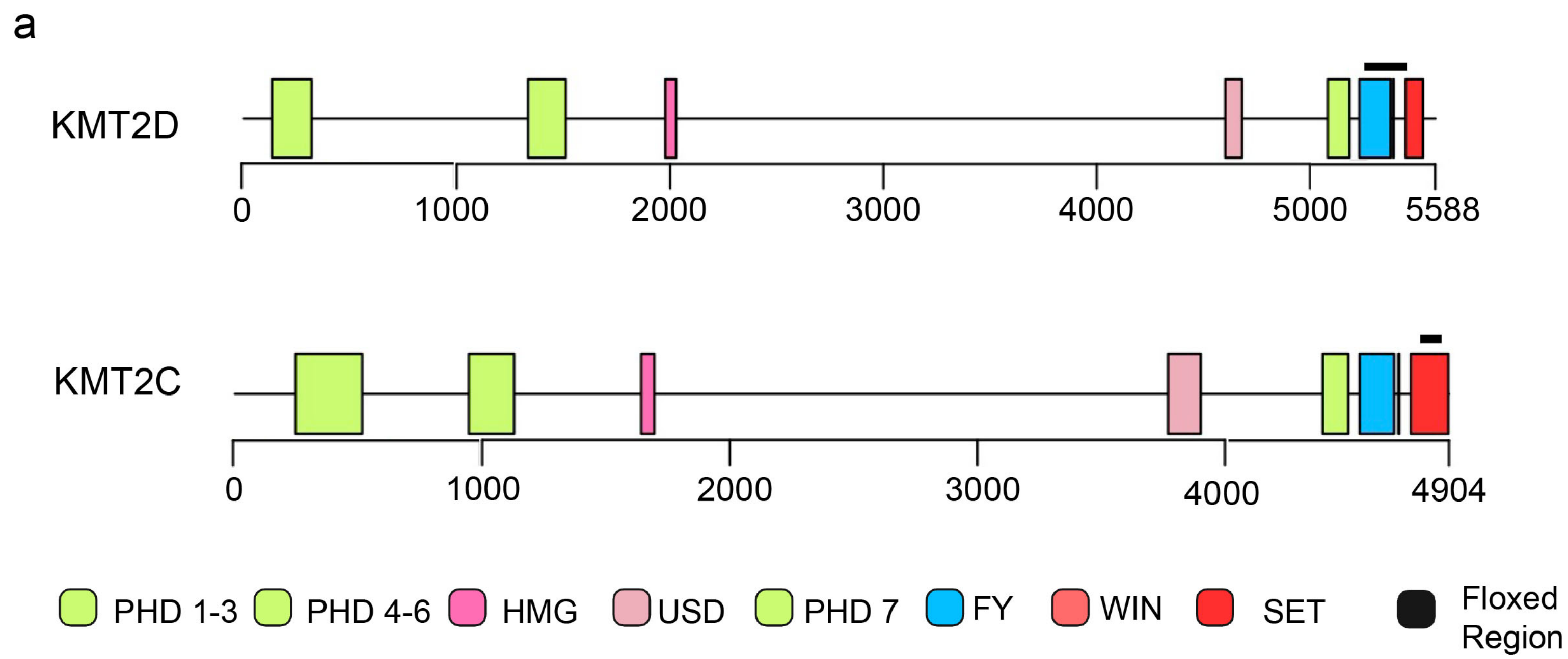

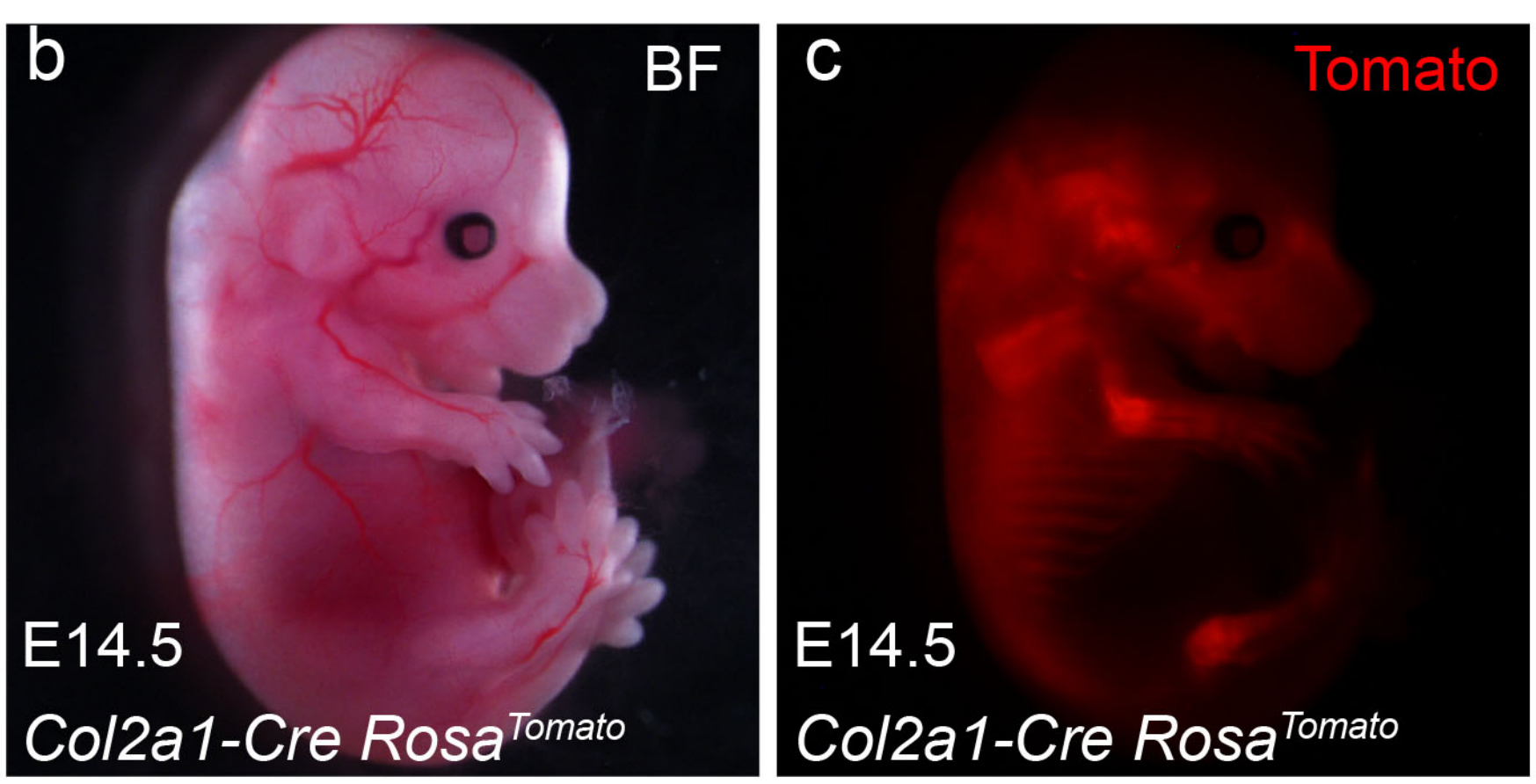

d

Genotype	Abbreviation
$Kmt2c^{fl/+}$ $Kmt2d^{fl/+}$ *Col2-Cre*	$C^{HET}D^{HET}$
$Kmt2c^{fl/fl}$ $Kmt2d^{fl/+}$ *Col2-Cre*	C^{KO}
$Kmt2c^{fl/+}$ $Kmt2d^{fl/fl}$ *Col2-Cre*	D^{KO}
$Kmt2c^{fl/fl}$ $Kmt2d^{fl/fl}$ *Col2-Cre*	$C^{KO}D^{KO}$

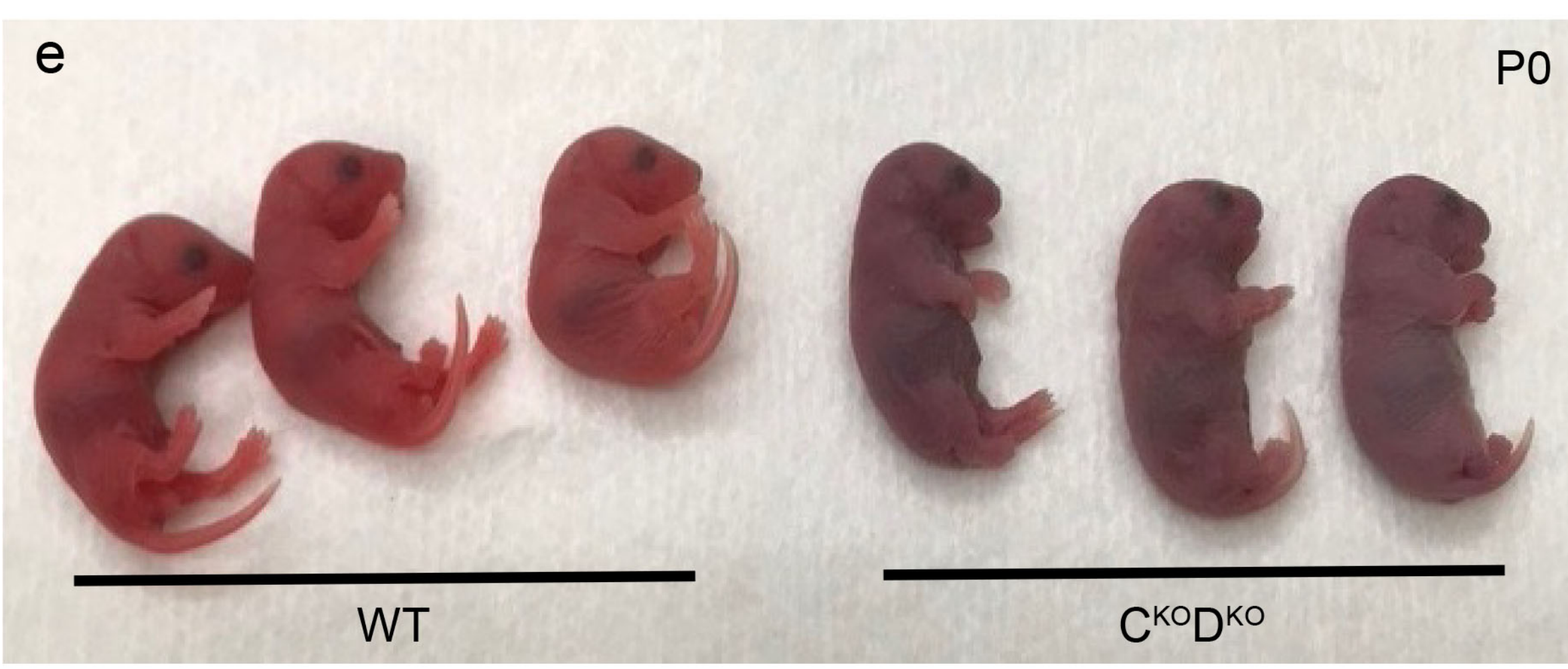

Figure S2

P24

Genotype	Observed	Expected	χ2	*p*
Wildtype	139	125.50	1.452	2.28×10^{-1}
$C^{HET}D^{HET}$	24	14.44	6.334	1.18×10^{-2}
C^{KO}	45	33.94	3.606	5.76×10^{-2}
D^{KO}	12	14.44	0.412	5.21×10^{-1}
$C^{KO}D^{KO}$	8	33.94	19.823	8.50×10^{-6}

P0

Genotype	Observed	Expected	χ2	*p*
Wildtype	100	95.00	0.263	6.10×10^{-1}
$C^{KO}D^{KO}$	7	29.88	17.515	2.85×10^{-5}

E18.5

Genotype	Observed	Expected	χ2	*p*
Wildtype	8	11.00	0.818	3.66×10^{-1}
$C^{KO}D^{KO}$	6	5.50	0.045	8.32×10^{-1}

Figure S3

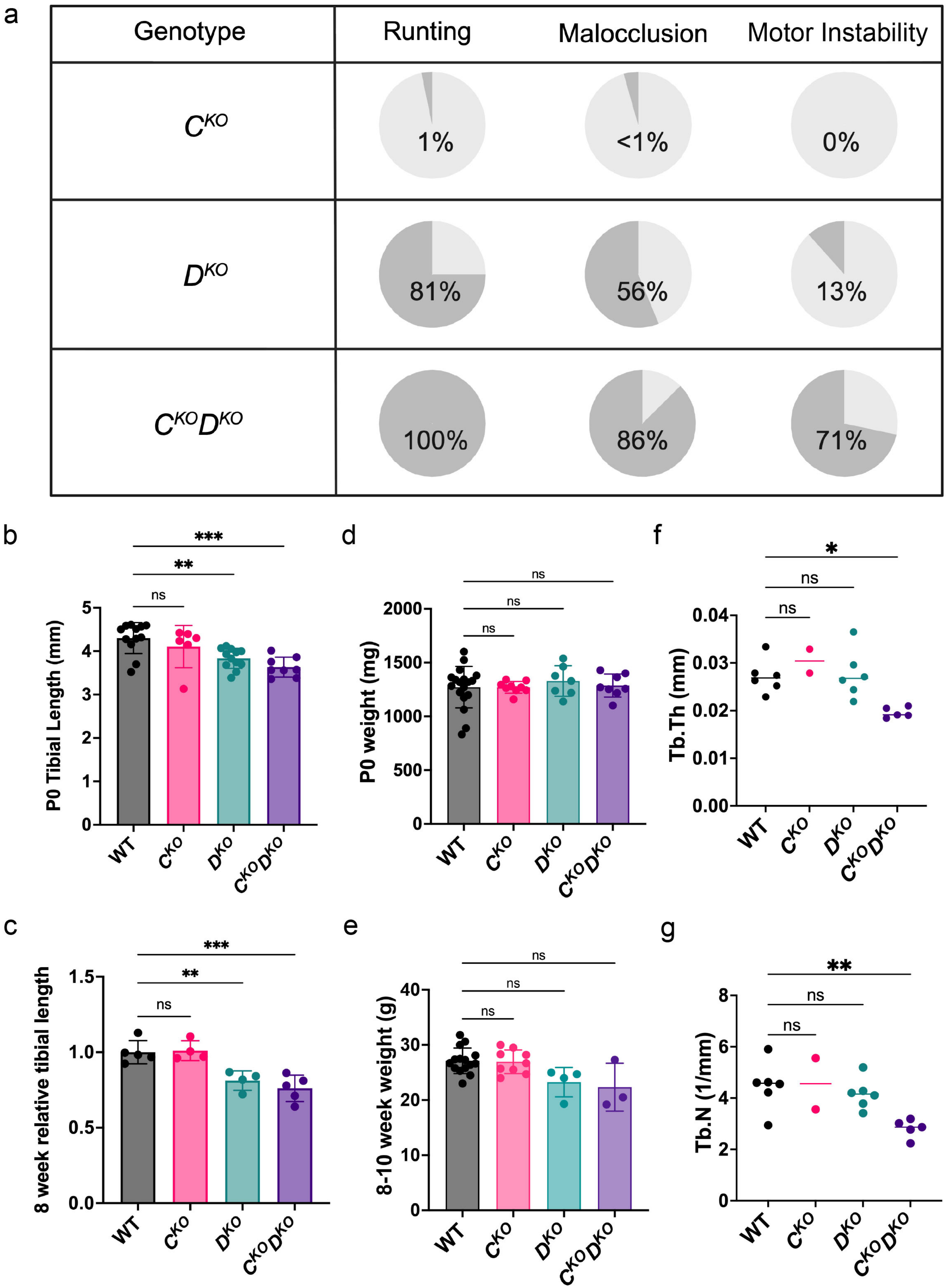

a
Genotype
Runting
Malocclusion
Motor Instability
C^{KO}
1%
<1%
0%
D^{KO}
81%
56%
13%
$C^{KO}D^{KO}$
100%
86%
71%
b
P0 Tibial Length (mm)
ns
**
***
WT
C^{KO}
D^{KO}
$C^{KO}D^{KO}$
c
8 week relative tibial length
ns
**
***
d
P0 weight (mg)
ns
e
8-10 week weight (g)
ns
f
Tb.Th (mm)
ns
*
g
Tb.N (1/mm)
ns
**

Figure S4

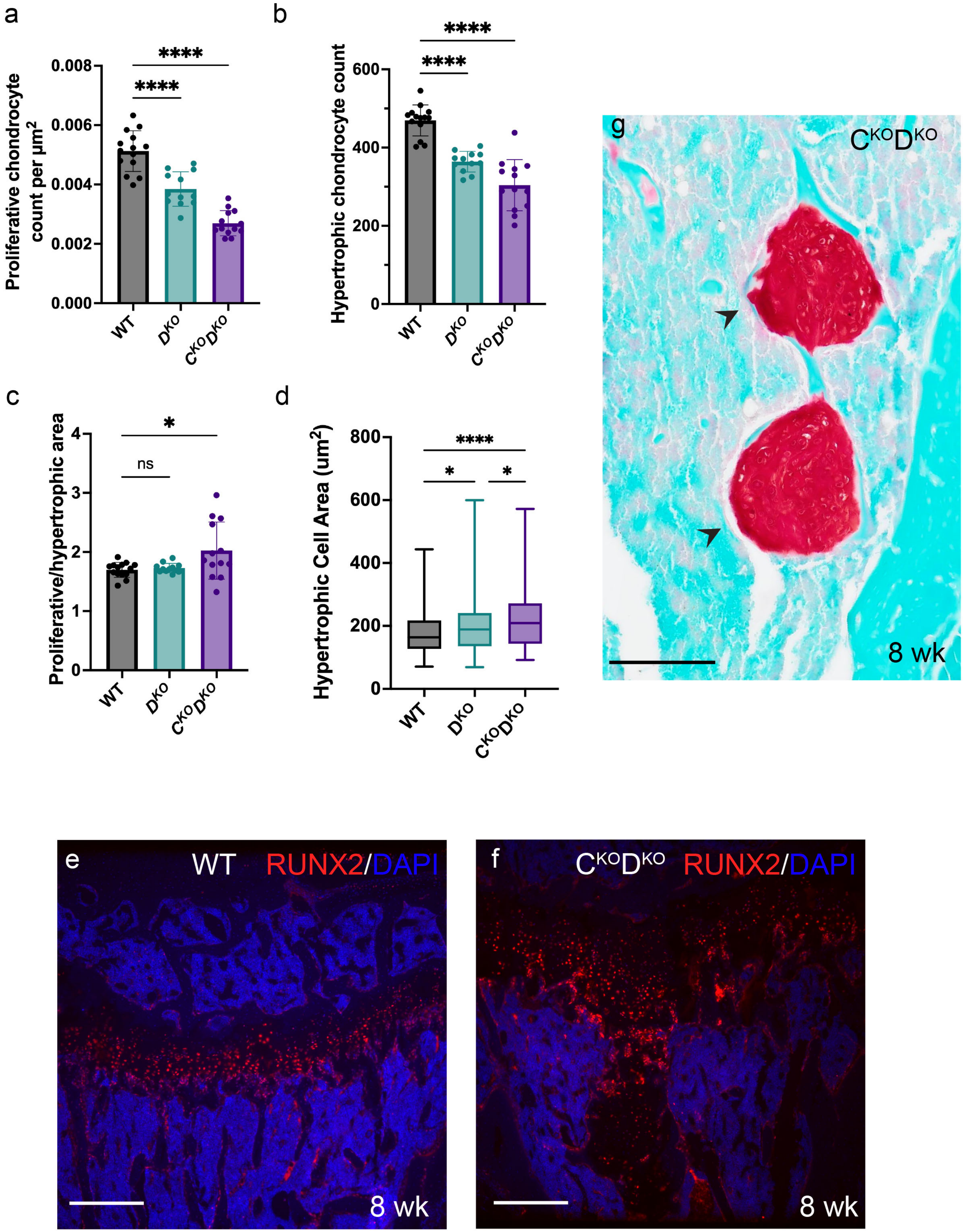

a
Proliferative chondrocyte count per μm²
0.008
0.006
0.004
0.002
0.000
****
****
WT
DKO
CKODKO
b
Hypertrophic chondrocyte count
600
400
200
0
****
****
WT
DKO
CKODKO
c
Proliferative/hypertrophic area
4
3
2
1
0
*
ns
WT
DKO
CKODKO
d
Hypertrophic Cell Area (um²)
800
600
400
200
0
****
*
*
WT
DKO
CKODKO
g
CKODKO
8 wk
e
WT RUNX2/DAPI
8 wk
f
CKODKO RUNX2/DAPI
8 wk

Figure S5

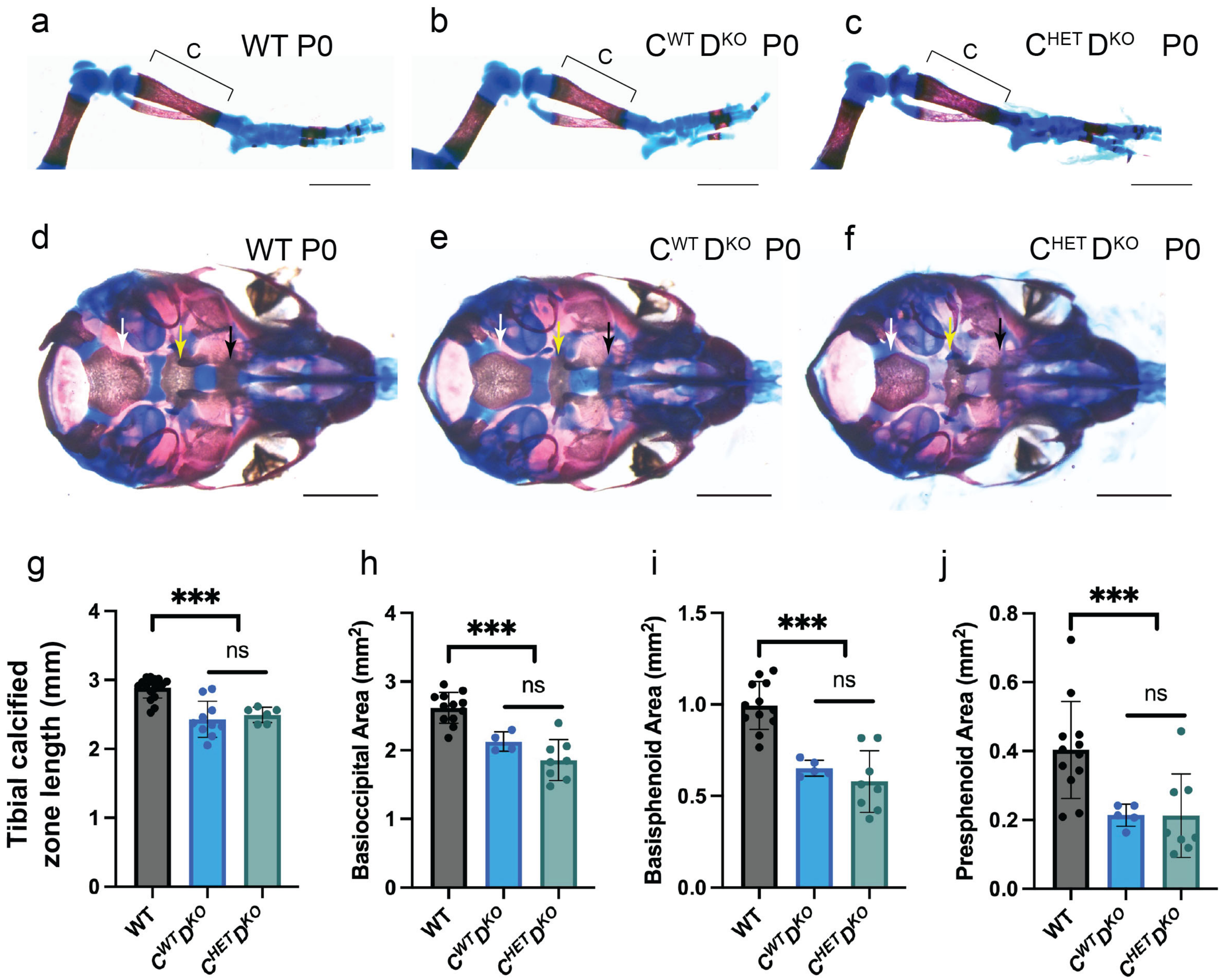

a
C
WT P0
b
C
C^WT D^KO P0
c
C
C^HET D^KO P0
d
WT P0
e
C^WT D^KO P0
f
C^HET D^KO P0
g
Tibial calcified zone length (mm)
***
ns
WT
C^WT D^KO
C^HET D^KO
h
Basioccipital Area (mm2)
***
ns
i
Basisphenoid Area (mm2)
***
ns
j
Presphenoid Area (mm2)
***
ns

Figure S6

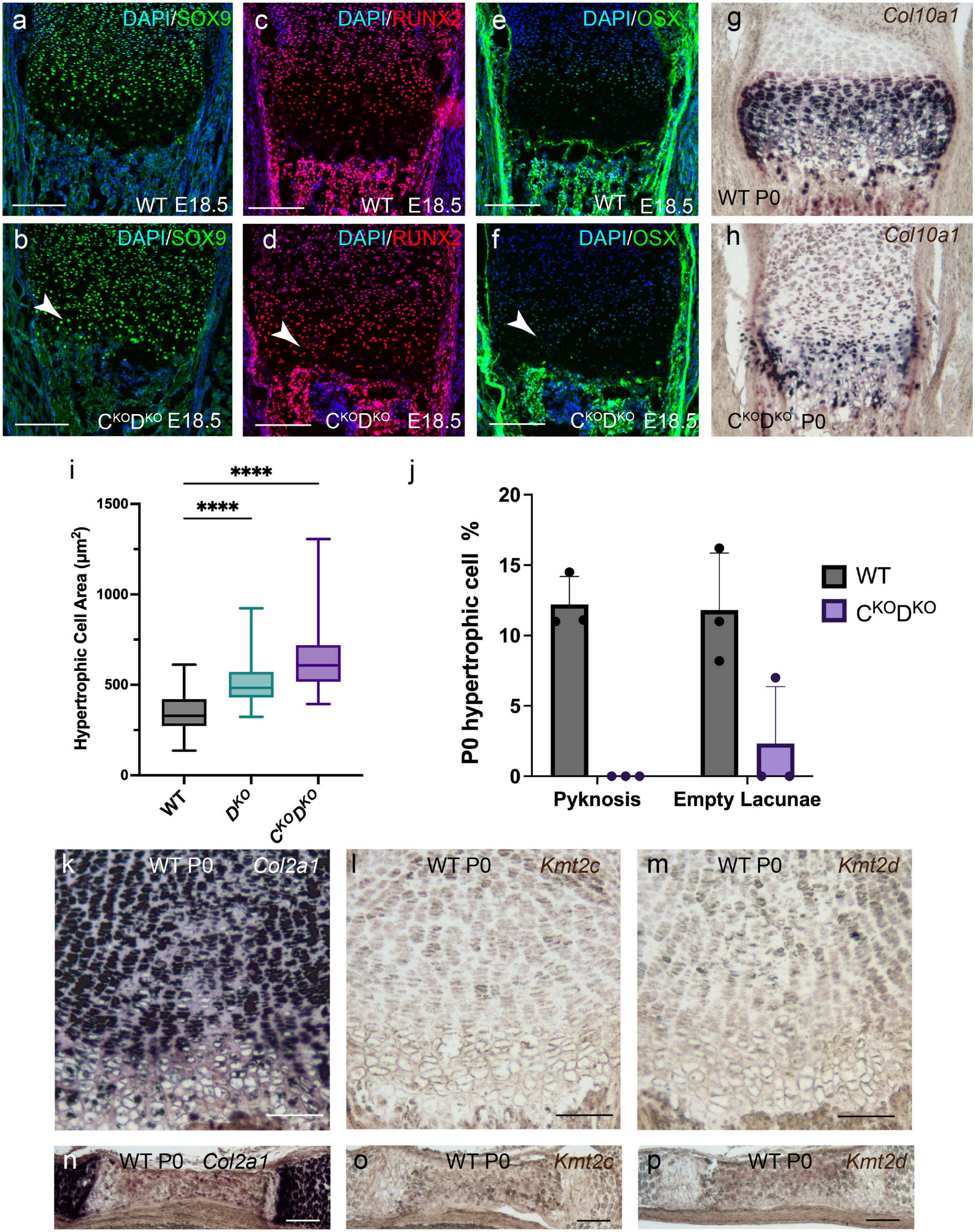
a
DAPI/SOX9
WT E18.5
b
DAPI/SOX9
C^KO D^KO E18.5
c
DAPI/RUNX2
WT E18.5
d
DAPI/RUNX2
C^KO D^KO E18.5
e
DAPI/OSX
WT E18.5
f
DAPI/OSX
C^KO D^KO E18.5
g
Col10a1
WT P0
h
Col10a1
C^KO D^KO P0
i
****
****
Hypertrophic Cell Area (µm2)
1500
1000
500
0
WT
D^KO
C^KO D^KO
j
P0 hypertrophic cell %
20
15
10
5
0
Pyknosis
Empty Lacunae
WT
C^KO D^KO
k
WT P0
Col2a1
l
WT P0
Kmt2c
m
WT P0
Kmt2d
n
WT P0
Col2a1
o
WT P0
Kmt2c
p
WT P0
Kmt2d

Figure S7

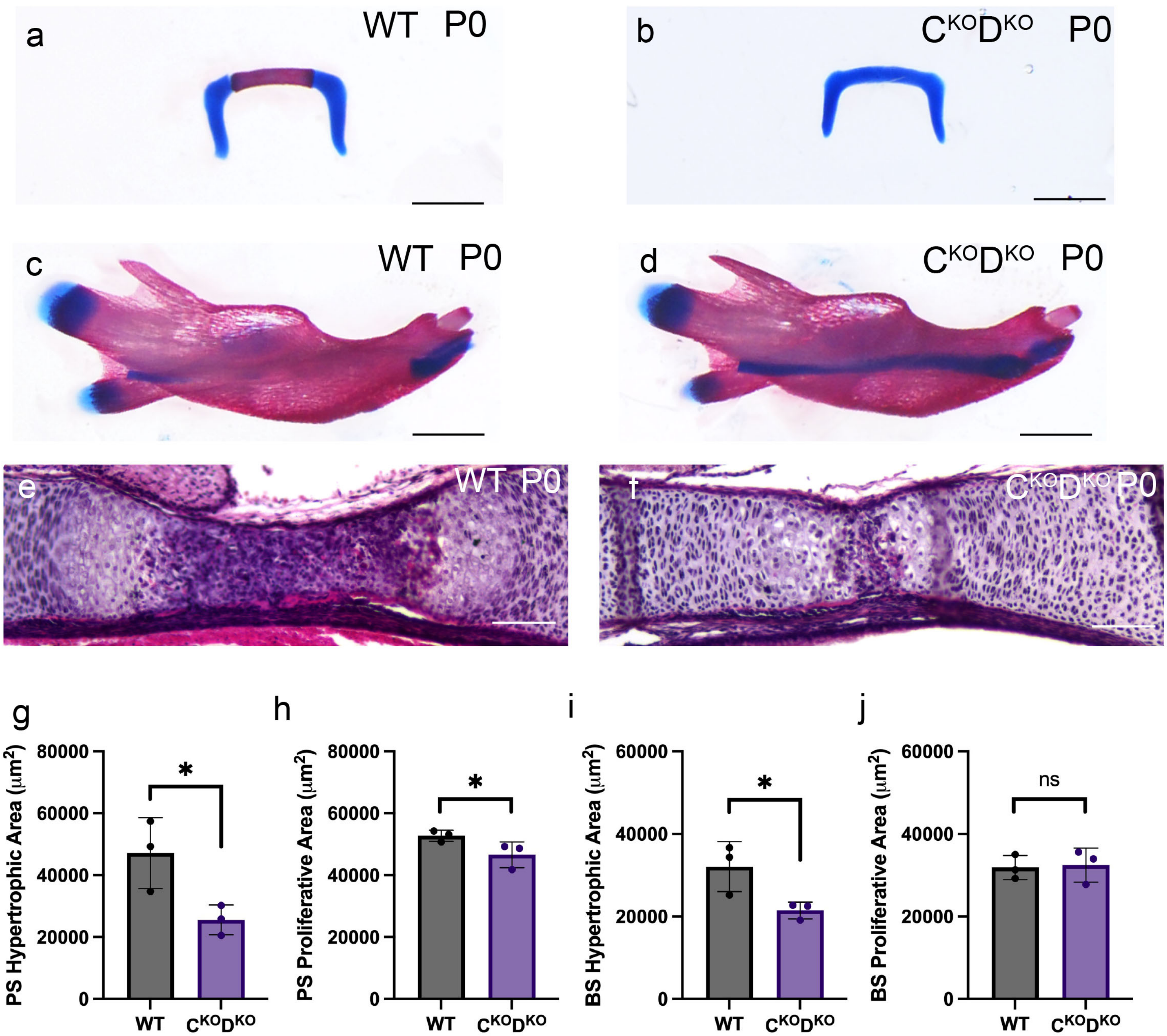
a
WT P0
b
C^KO D^KO P0
c
WT P0
d
C^KO D^KO P0
e
WT P0
f
C^KO D^KO P0
g
PS Hypertrophic Area (μm²)
80000
60000
40000
20000
0
*
WT
C^KO D^KO
h
PS Proliferative Area (μm²)
80000
60000
40000
20000
0
*
WT
C^KO D^KO
i
BS Hypertrophic Area (μm²)
60000
40000
20000
0
*
WT
C^KO D^KO
j
BS Proliferative Area (μm²)
60000
40000
20000
0
ns
WT
C^KO D^KO

Figure S8

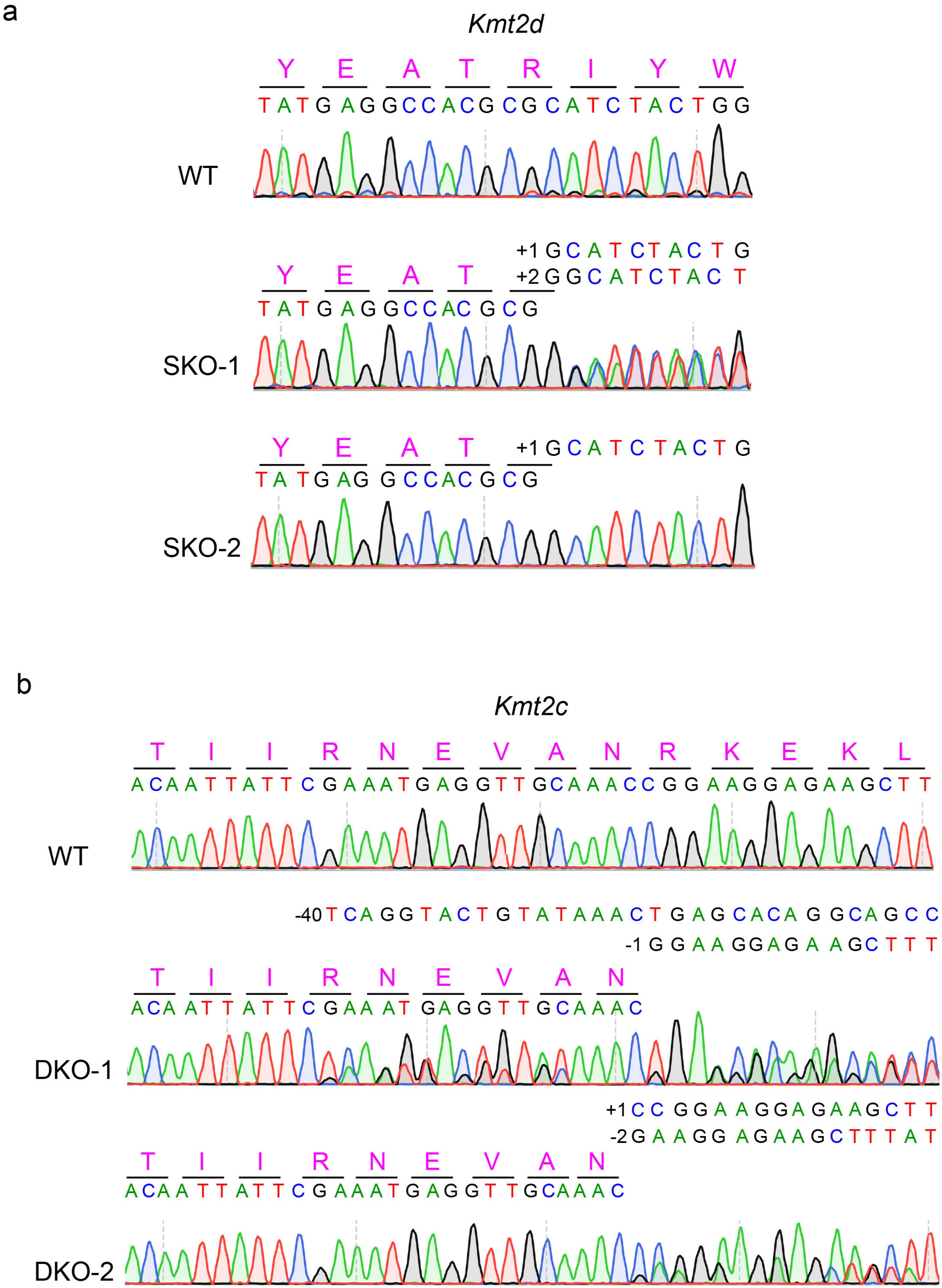
a
Kmt2d
Y E A T R I Y W
TATGAGGCCACGCGCATCTACTGG
WT
+1 GCATCTACTG
+2 GGCATCTACT
Y E A T
TATGAGGCCACGCG
SKO-1
Y E A T +1 GCATCTACTG
TATGAGGCCACGCG
SKO-2
b
Kmt2c
T I I R N E V A N R K E K L
ACAATTATTCGAAATGAGGTTGCAAACCGGAAGGAGAAGCTT
WT
-40 TCAGGTACTGTATAAACTGAGCACAGGCAGCC
-1 GGAAGGAGAAGCTTT
T I I R N E V A N
ACAATTATTCGAAATGAGGTTGCAAAC
DKO-1
+1 CCGGAAGGAGAAGCTT
-2 GAAGGAGAAGCTTTAT
T I I R N E V A N
ACAATTATTCGAAATGAGGTTGCAAAC
DKO-2

Figure S9

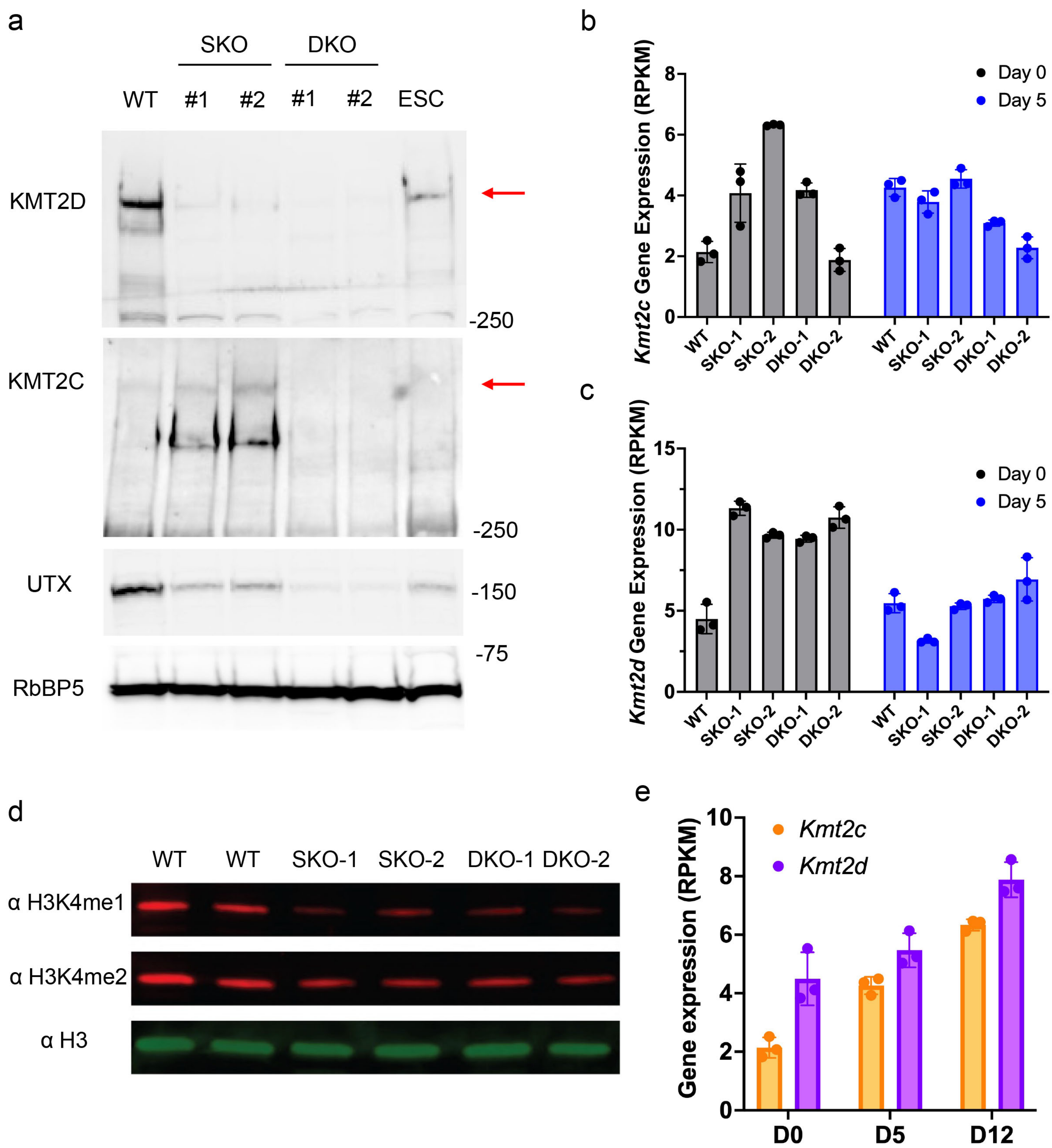

a
SKO
DKO
WT
#1
#2
#1
#2
ESC
KMT2D
KMT2C
UTX
RbBP5
-250
-250
-150
-75
b
Kmt2c Gene Expression (RPKM)
Day 0
Day 5
WT
SKO-1
SKO-2
DKO-1
DKO-2
c
Kmt2d Gene Expression (RPKM)
Day 0
Day 5
d
WT
WT
SKO-1
SKO-2
DKO-1
DKO-2
α H3K4me1
α H3K4me2
α H3
e
Kmt2c
Kmt2d
Gene expression (RPKM)
D0
D5
D12

Figure S10

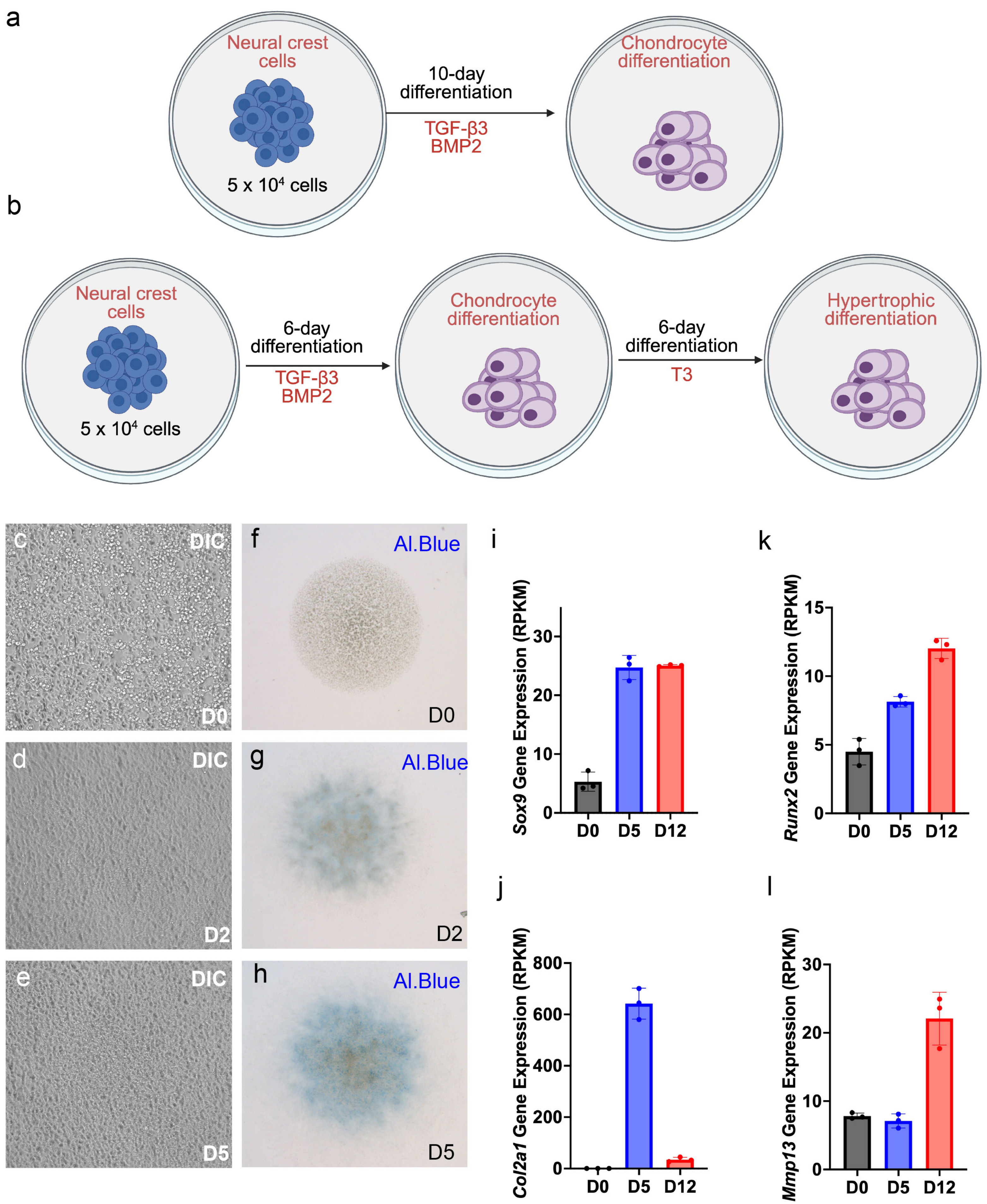
a
Neural crest cells
5 x 10⁴ cells
10-day differentiation
TGF-β3
BMP2
Chondrocyte differentiation
b
Neural crest cells
5 x 10⁴ cells
6-day differentiation
TGF-β3
BMP2
Chondrocyte differentiation
6-day differentiation
T3
Hypertrophic differentiation
c
DIC
D0
d
DIC
D2
e
DIC
D5
f
Al.Blue
D0
g
Al.Blue
D2
h
Al.Blue
D5
i
Sox9 Gene Expression (RPKM)
0
10
20
30
D0
D5
D12
k
Runx2 Gene Expression (RPKM)
0
5
10
15
D0
D5
D12
j
Col2a1 Gene Expression (RPKM)
0
200
400
600
800
D0
D5
D12
l
Mmp13 Gene Expression (RPKM)
0
10
20
30
D0
D5
D12

Figure S11

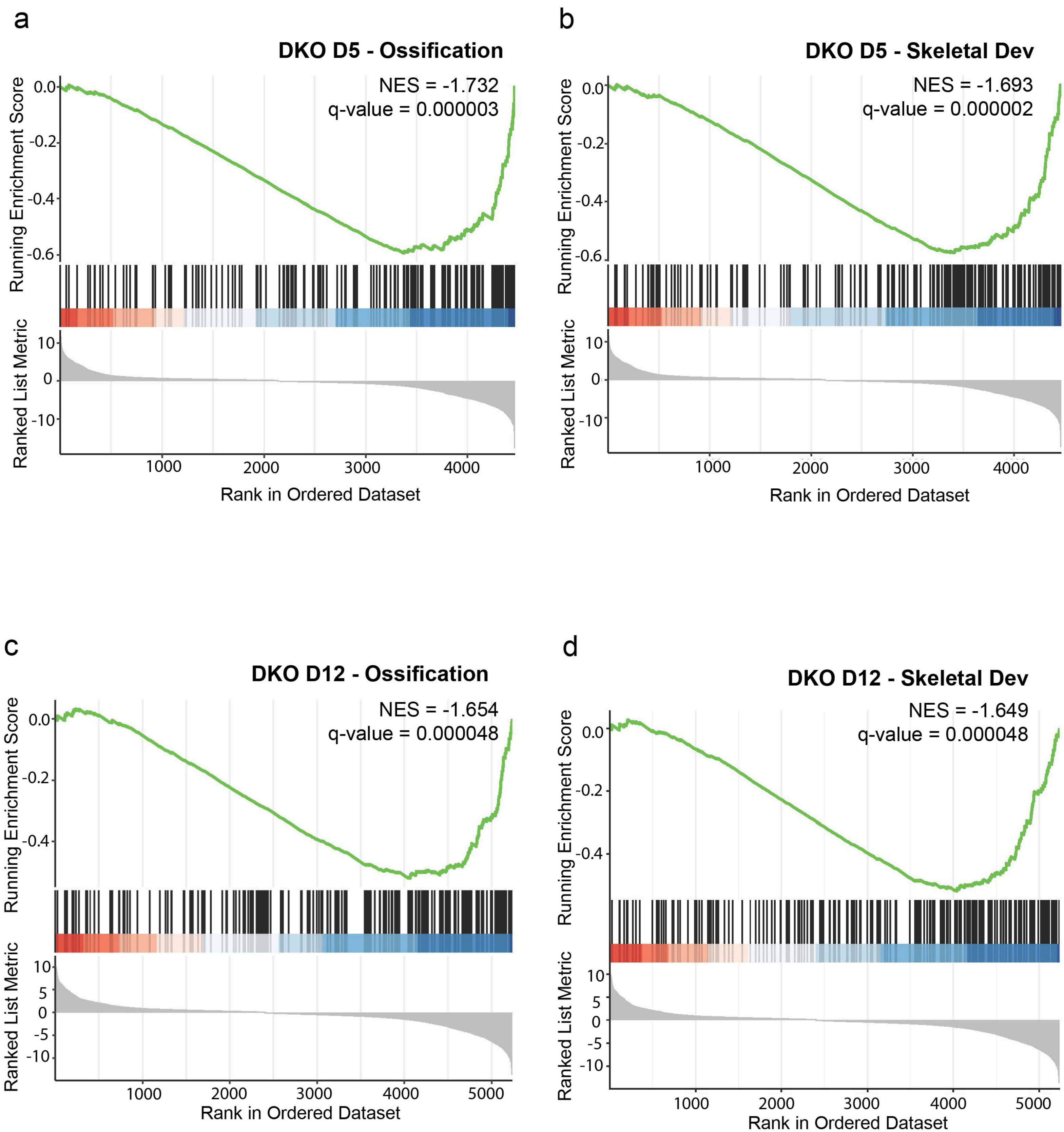
a
DKO D5 - Ossification
NES = -1.732
q-value = 0.000003
Running Enrichment Score
0.0
-0.2
-0.4
-0.6
Ranked List Metric
10
0
-10
1000
2000
3000
4000
Rank in Ordered Dataset
b
DKO D5 - Skeletal Dev
NES = -1.693
q-value = 0.000002
Running Enrichment Score
0.0
-0.2
-0.4
-0.6
Ranked List Metric
10
0
-10
1000
2000
3000
4000
Rank in Ordered Dataset
c
DKO D12 - Ossification
NES = -1.654
q-value = 0.000048
Running Enrichment Score
0.0
-0.2
-0.4
Ranked List Metric
10
5
0
-5
-10
1000
2000
3000
4000
5000
Rank in Ordered Dataset
d
DKO D12 - Skeletal Dev
NES = -1.649
q-value = 0.000048
Running Enrichment Score
0.0
-0.2
-0.4
Ranked List Metric
10
5
0
-5
-10
1000
2000
3000
4000
5000
Rank in Ordered Dataset

Figure S12

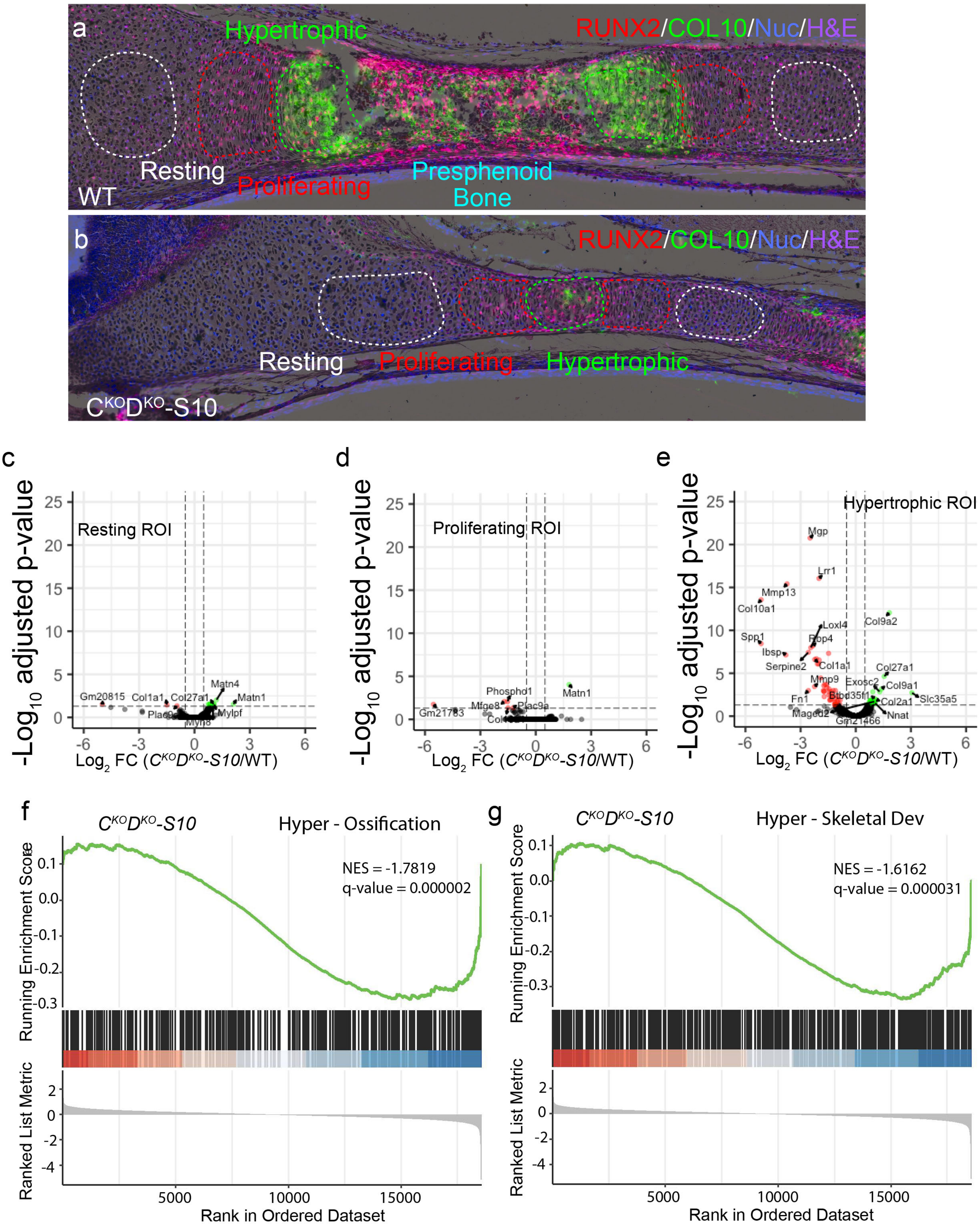
a
Hypertrophic
RUNX2/COL10/Nuc/H&E
Resting
Proliferating
Presphenoid Bone
WT
b
RUNX2/COL10/Nuc/H&E
Resting
Proliferating
Hypertrophic
CKODKO-S10
c
Resting ROI
-Log10 adjusted p-value
Log2 FC (CKODKO-S10/WT)
Gm20815
Col1a1
Col27a1
Matn4
Matn1
Mylpf
Plac9a
Myl6
d
Proliferating ROI
Phospho1
Matn1
Mfge8
Plac9a
Gm21783
e
Hypertrophic ROI
Mgp
Lrr1
Mmp13
Col10a1
Loxl4
Col9a2
Spp1
Rbp4
Ibsp
Serpine2
Col1a1
Col27a1
Mmp9
Exosc2
Col9a1
Fn1
Btbd35f1
Col2a1
Slc35a5
Maged2
Gm21466
Nnat
f
CKODKO-S10
Hyper - Ossification
NES = -1.7819
q-value = 0.000002
Running Enrichment Score
Ranked List Metric
Rank in Ordered Dataset
g
CKODKO-S10
Hyper - Skeletal Dev
NES = -1.6162
q-value = 0.000031
Running Enrichment Score
Ranked List Metric
Rank in Ordered Dataset

Figure S13

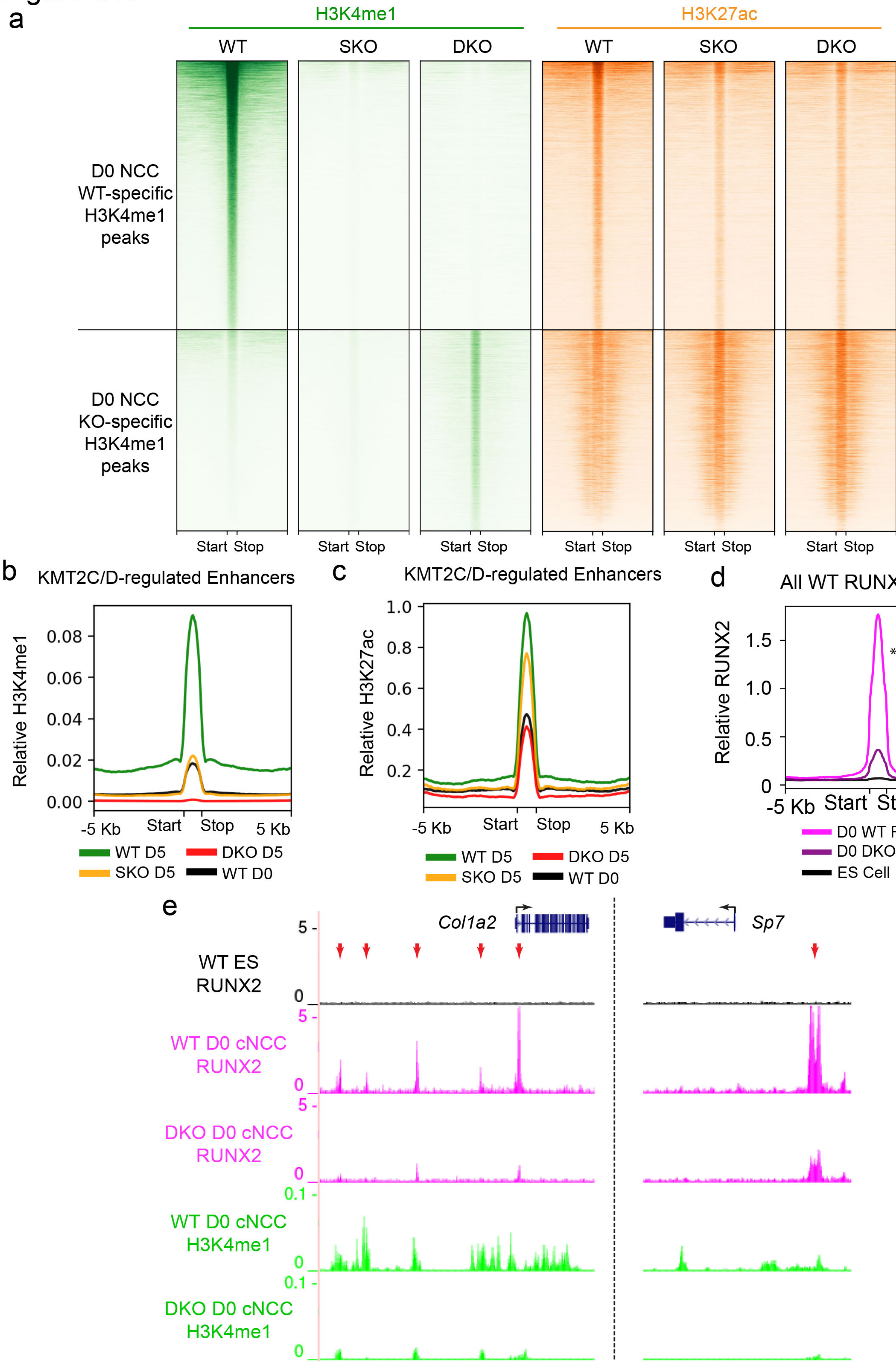
a
H3K4me1
H3K27ac
WT
SKO
DKO
WT
SKO
DKO
D0 NCC WT-specific H3K4me1 peaks
D0 NCC KO-specific H3K4me1 peaks
Start Stop
b
KMT2C/D-regulated Enhancers
Relative H3K4me1
0.08
0.06
0.04
0.02
0.00
-5 Kb
Start
Stop
5 Kb
WT D5
SKO D5
DKO D5
WT D0
c
KMT2C/D-regulated Enhancers
Relative H3K27ac
1.0
0.8
0.6
0.4
0.2
-5 Kb
Start
Stop
5 Kb
WT D5
SKO D5
DKO D5
WT D0
d
All WT RUNX2 peaks
Relative RUNX2
1.5
1.0
0.5
0
****
-5 Kb
Start
Stop
5 Kb
D0 WT RUNX2
D0 DKO RUNX2
ES Cell RUNX2
e
Col1a2
Sp7
WT ES RUNX2
WT D0 cNCC RUNX2
DKO D0 cNCC RUNX2
WT D0 cNCC H3K4me1
DKO D0 cNCC H3K4me1
5 -
0
0.1 -
