# Supplementary material for: KMT2C and KMT2D regulate skeletal development through stage-specific epigenetic control of chondrogenesis
Source: Bone Res. 2026 Oct 27;14:107. doi: 10.1038/s41413-026-00584-8 (PMC13714318; doi:10.1038/s41413-026-00584-8)
Supplement: Supplementary file 2 [file 41413_2026_584_MOESM1_ESM.docx]

**Supplemental Figures**

**Figure S1:** **a** Schematic of KMT2C and KMT2D proteins with locations of PHD fingers (PHD), High Mobility Group domain (HMG), UTX Stabilization Domain (USD), FY-rich domain (FY), WW domain-interacting domain (WIN), and Suppressor of variegation, Enhancer of zeste, and Trithorax domain (SET). *In vivo* floxed regions annotated by black bars (exon 50-51 in KMT2D and exon 56-58 in KMT2C). **b-c** Representative whole-mount images of E14.5 *Col2a1-Cre; Rosa^Tomato^* reporter embryo. Brightfield (BF) is shown on the left and Tomato fluorescence is shown on the right. **d** Genotype abbreviations for *Col2a1-Cre* mouse model. **e** Partial penetrance of *C^KO^D^KO^* pup discoloration as compared to WT at P0.

**Figure S2:** Chi square analysis of offspring from *Kmt2c (fl/fl) Kmt2d (fl/fl) x Kmt2c (fl/+) Kmt2d (fl/+) Col2a1-Cre* crosses at P24, P0, and E18.5. *C^KO^D^KO^* exhibit significant reductions at weaning (P24) and birth (P0).

**Figure S3:** **a** Frequency of runting, malocclusion, and motor instability observed in weaning age (P24) WT, *C^KO^*, *D^KO^*, and *C^KO^D^KO^* mice (*N=*36, *N=*16, *N=*14, *N=*7). **b** Tibial bone length (mm) of P0 WT, *C^KO^*, *D^KO^*, and *C^KO^D^KO^* mice (N=12, N=6, N=12, N=8 respectively). **c** Relative tibial bone length of 8-week WT, *C^KO^*, *D^KO^*, and *C^KO^D^KO^* mice (N=5, N=4, N=4, N=5 respectively). **d** Weight (mg) of P0 WT, *C^KO^*, *D^KO^*, and *C^KO^D^KO^* newborn pups (N=18, N=9, N=7, N=8 respectively). **e** Weight (g) of 8-10 week male WT, *C^KO^*, *D^KO^*, and *C^KO^D^KO^* mice (N=15, N=9, N=4, N=3 respectively). **f-g** Micro-CT analysis of trabecular bone formation (f: Tb.Th, g: Tb.N) in 8-week-old tibia of WT, *C^KO^*, *D^KO^*, and *C^KO^D^KO^* mice (N=6, N=2, N=6, N=5, respectively). Data shown are mean ± SD. Statistical analyses were performed with ordinary one-way ANOVA followed by Dunnett’s test. *P<0.05, **P<0.01, ***P<0.001, ****P<0.0001, ns = not significant where specified.

**Figure S4: a-d** The density of proliferative chondrocytes per µm^2^ (a), the total number of hypertrophic chondrocytes per section (b), the ratio of proliferative to hypertrophic area (c), and individual hypertrophic cell area in µm^2^ (d) in 8-week-old proximal tibial growth plates of WT, *D^KO^*, and *C^KO^D^KO^* (N=4, N=3, N=4, respectively). **e-f** Immunofluorescence of RUNX2 with DAPI in 8-week-old coronally sectioned proximal tibial growth plates of WT and *C^KO^D^KO^* indicating disorganization of *C^KO^D^KO^* growth plate and penetrance of RUNX2-expressing cells into the diaphysis. Scale bar is 250 µm. **g** Safranin O/fast green staining in the tibial diaphysis of *C^KO^D^KO^*. Illustrated is the same region of RUNX2-expressing cells as shown in parts e-f*.* Scale bar is 200 µm. Data shown are mean ± SD. Statistical analyses were performed using ordinary one-way ANOVA followed by Dunnett’s test *P<0.05, **P<0.01, ***P<0.001, ****P<0.0001, ns = not significant where specified.

**Figure S5: a-c** Alcian blue and alizarin red staining for cartilage and bone of WT, *C^WT^D^KO^*, and *C^HET^D^KO^* P0 hindlimbs. Calcified region (c) demarcated with brackets. Scale bar is 1 mm. **d-f** P0 alizarin red and alcian blue staining of WT, *C^WT^D^KO^*, and *C^HET^D^KO^* with ventral whole mount view of the cranial base. Basioccipital (white arrow), basisphenoid (yellow arrow), and presphenoid (black arrow) are indicated. Scale bar is 1 mm. **g** Length (mm) of calcified region of bone in P0 WT, *C^WT^D^KO^*, and *C^HET^D^KO^* tibia (*N*=12, *N*=10, *N*=6, respectively). **h-j** Area (mm^2^) of basioccipital (BO), basisphenoid (BS), and presphenoid (PS) regions in P0 WT, *C^WT^D^KO^*, and *C^HET^D^KO^* cranial base (*N*=12, *N*=4, *N*=8, respectively). Data shown are mean ± SD. Statistical analyses were performed using ordinary one-way ANOVA followed by Dunnett’s test *P<0.05, **P<0.01, ***P<0.001, ****P<0.0001, ns = not significant where specified.

**Figure S6:** **a-f** Immunofluorescence for SOX9 (green) (a-b), RUNX2 (red) (c-d), or osterix (OSX, green) (e-f) in E18.5 proximal tibial growth plate of WT and *C^KO^D^KO^* embryos. White arrows indicate altered hypertrophic zone in *C^KO^D^KO^*. Scale bar is 200 µm. **g-h** Brightfield (BF) imaging of *in situ* hybridization for *Col10a1* in P0 proximal tibial growth plate of WT and *C^KO^D^KO^* embryos. *Col10a1* expression was consistently downregulated in *C^KO^D^KO^* (N=3). **i** Individual hypertrophic cell area (µm^2^) in P0 proximal tibial growth plates of WT, *D^KO^*, and *C^KO^D^KO^* (*N*=8, *N*=3, *N*=3, respectively). **j** Quantitation of the percentage of hypertrophic chondrocytes displaying pyknosis or empty lacunae (N=3). See Fig. 3n for examples. **k-p** Brightfield imaging of RNA *in situ* hybridization for *Col2a1*, *Kmt2c*, and *Kmt2d* in P0 proximal tibial growth plates (k-m) or presphenoid (PS) region of cranial base (n-p). Scale bars = 100 µm. Data shown are mean ± SD. Statistical analyses were performed using ordinary one-way ANOVA followed by Dunnett’s test *P<0.05, **P<0.01, ***P<0.001, ****P<0.0001, ns = not significant where specified.

**Figure S7: a-d** Whole mount images of alizarin red and alcian blue stained dissected hyoid cartilage (a-b) and mandible (c-d) in WT and *C^KO^D^KO^* P0 mice. Scale bar is 1 mm. **e-f** Hematoxylin and eosin (H&E) staining of P0 sagittal section of presphenoid (PS) region of bone in WT and *C^KO^D^KO^* mice. Scale bar is 100 µm. **g-j** Area (µm^2^) of PS and BS hypertrophic (g and i, respectively) and proliferative area (h and j) in WT and *C^KO^D^KO^* P0 mice (N = 3 per genotype). Data shown are mean ± SD. Statistical analyses were performed using ordinary one-way ANOVA followed by Dunnett’s test *P<0.05, **P<0.01, ***P<0.001, ****P<0.0001, ns = not significant where specified.

**Figure S8: a** Sanger sequencing chromatograms of the *Kmt2d* locus in WT, SKO-1, and SKO-2 cell lines with frameshift mutations indicated (e.g. heterozygous +1 bp and +2bp in SKO-1). Shift in open reading frame (ORF) indicated by the divergence in amino acid translations (pink) relative to WT. **b** Sanger sequencing chromatograms of the *Kmt2c* locus in WT, DKO-1, and DKO-2 cell lines with frameshift mutations indicated (e.g. -40 bp and -1bp in DKO-1). Shift in open reading frame (ORF) indicated by the divergence in amino acid translations (pink) relative to WT.

**Figure S9:** **a** Western blot for KMT2D, KMT2C, UTX, and RbBP5 control antibodies in undifferentiated cNCCs in WT, SKO line 1 (SKO #1), SKO line 2 (SKO #2), DKO line 1 (DKO #1) and DKO line 2 (DKO #2) with reference to mouse embryonic stem cell (ESC) lysate. **b-c** *Kmt2c* and *Kmt2d* gene expression (RPKM) at Day 0 (black) and Day 5 (blue) of chondrocyte differentiation in WT, SKO-1, SKO-2, DKO-1, and DKO-2. **d** Western blot for H3K4me1 (top), H3K4me2 (middle), and H3 (bottom) control antibodies in undifferentiated cNCCs in WT, SKO line 1 (SKO-1), SKO line 2 (SKO-2), DKO line 1 (DKO-1) and DKO line 2 (DKO-2). **e** WT expression level (RPKM) of *Kmt2c* (orange) and *Kmt2d* (purple) at Day 0, Day 5, and Day 12 of chondrogenic differentiation. Data shown are mean ± SD.

**Figure S10:** **a-b** Differentiation model for cNCC *in vitro* culture depicting 10-day chondrogenic differentiation (a) and 12-day hypertrophic differentiation (b). Model created with BioRender.com. **c-h** DIC imaging and alcian blue staining for proteoglycan production in WT at Day 0 (D0), Day 2 (D2), and Day 5 (D5) of chondrogenic differentiation. **i-l** WT expression levels (RPKM) of *Sox9* (i), *Col2a1* (j), *Runx2* (k), and *Mmp13* (l) at Day 0 (gray), Day 5 chondrogenic (blue), and Day 12 hypertrophic (red) differentiation. Data shown are mean ± SD.

**Figure S11:** **a-d** GSEA of all dysregulated genes in DKO as compared to WT at Day 5 (D5) (a-b) or Day 12 (D12) (c-d) of differentiation for “Ossification” and “Skeletal System Development” gene sets.

**Figure S12:** **a-b** Sagittal sections of presphenoid bone collected for spatial transcriptomic analysis in WT (a) and *C^KO^D^KO^-S10* (b). Hypertrophic (green), proliferative (red), and resting (white) regions collected for analysis demarcated by dashed regions. RUNX2 (red) and COL10A1 (green), DAPI (blue), and H&E staining shown. **c-e** Volcano plots of log_2_FC of *C^KO^D^KO^-S10* differentially expressed genes from the resting (c), proliferative (d), or hypertrophic (e) regions relative to WT. Genes with FDR < 0.05 and log_2_FC > 1 or < -1 are demarcated in green or red, respectively. **f-g** GSEA of dysregulated hypertrophic region genes in *C^KO^D^KO^-S10* as compared to WT for “Ossification” (f) and “Skeletal System Development” (g) gene sets.

**Figure S13:** **a** Heatmap of H3K4me1 (green) and H3K27ac (orange) CUT&Tag peaks in Day 0 undifferentiated WT, SKO, and DKO lines, classified by peaks of H3K4me1 that are WT-specific or those that are KO-specific (found only in either SKO or DKO lines). **b-c** Plot profiles of H3K4me1 (b) or H3K27ac (c) levels at all KMT2C/D-regulated enhancers. Depicted are respective levels at WT Day 0 (black), WT Day 5 chondrogenic (green), SKO Day 5 (orange), and DKO Day 5 (red). ± 5 Kb up- and downstream of enhancer peaks. **d** Plot profile of RUNX2 CUT&RUN relative enrichment at all RUNX2 peaks for comparison of WT cNCCs (pink), DKO cNCCs (purple), and negative control ES cells (black). Asterisks denote significant read enrichment in WT compared to DKO across peaks (*t*-test *p* < 0.0001). **e** UCSC genome browser tracks of RUNX2 CUT&RUN (pink) and H3K4me1 (green) at two RUNX2 target genes (*Col1a2* and *Sp7*) in WT and DKO cNCCs. ES cell RUNX2 CUT&RUN (black) serves as a negative control. RUNX2 peaks lost in DKO cNCCs are denoted by red arrows.

**Table S1:** All genes experiencing significant (FDR < 0.05 and logFC < -1) loss of expression in both SKO lines relative to WT at D0 (undifferentiated cNCC).

**Table S2:** All genes experiencing significant (FDR < 0.05 and logFC < -1) loss of expression in both DKO lines relative to WT at D0 (undifferentiated cNCC).

**Table S3:** All genes experiencing significant (FDR < 0.05 and logFC < -1) loss of expression in both SKO lines relative to WT at D5 of chondrogenic differentiation.

**Table S4:** All genes experiencing significant (FDR < 0.05 and logFC < -1) loss of expression in both DKO lines relative to WT at D5 of chondrogenic differentiation.

**Table S5:** All genes experiencing significant (FDR < 0.05 and logFC < -1) loss of expression in both SKO lines relative to WT at D12 of hypertrophic chondrocyte differentiation.

**Table S6:** All genes experiencing significant (FDR < 0.05 and logFC < -1) loss of expression in both DKO lines relative to WT at D12 of hypertrophic chondrocyte differentiation.

**Table S7:** All D5 enhancers regulated by KMT2C and KMT2D experiencing loss of H3K4me1 in DKO at D5 of chondrogenic differentiation. This enhancer set was utilized for chromatin profiling (Fig. S13b-c).

**Table S8:** All enhancers and regulated skeletal genes (ABC computationally predicted) experiencing loss of expression in DKO at D5 of chondrogenic differentiation. This enhancer set was utilized for chromatin profiling (Fig. 6g-j).

**Table S9:** All WT D0 (cNCC) RUNX2 peaks with average WT and DKO RPKM values, log fold change (logFC) and significance (FDR). Scaling factor calculations from spike in *E. coli* reads are illustrated in sheet 2.
